# 2,3-Dihydroquinazolin-4(1*H*)-one as a privileged scaffold in drug design

**DOI:** 10.1039/c8ra02827c

**Published:** 2018-06-07

**Authors:** Mariateresa Badolato, Francesca Aiello, Nouri Neamati

**Affiliations:** Department of Pharmacy, Health and Nutritional Sciences, University of Calabria Ed. Polifunzionale 87036 Arcavacata di Rende CS Italy; Department of Medicinal Chemistry, College of Pharmacy, University of Michigan, North Campus Research Complex 1600 Huron Parkway Ann Arbor MI 48109 USA

## Abstract

2,3-Dihydroquinazolin-4-one (DHQ) belongs to the class of nitrogen-containing heterocyclic compounds representing a core structural component in various biologically active compounds. In the past decades, several methodologies have been developed for the synthesis of the DHQ framework, especially the 2-substituted derivatives. Unfortunately, multistep syntheses, harsh reaction conditions, and the use of toxic reagents and solvents have limited their full potential as a versatile fragment. Recently, use of green chemistry and alternative strategies are being explored to prepare diverse DHQ derivatives. This fragment is used as a synthon for the preparation of biologically active quinazolinones and as a functional substrate for the synthesis of modified DHQ derivatives exhibiting different biological properties. In this review, we provide a comprehensive assessment of the synthesis and biological evaluations of DHQ derivatives.

## Abstract 2

2,3-Dihydroquinazolin-4-one (DHQ) belongs to the class of nitrogen-containing heterocyclic compounds representing a core structural component in various biologically active compounds.

## Graphical abstract

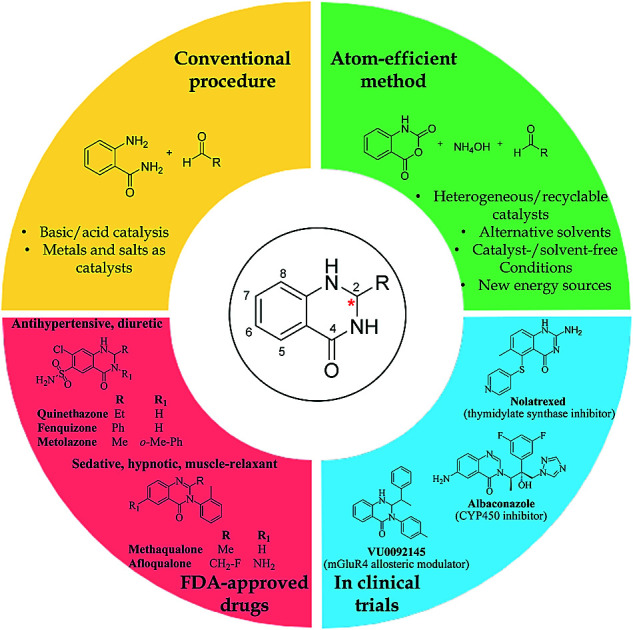

## Introduction

**Fig. 1:**
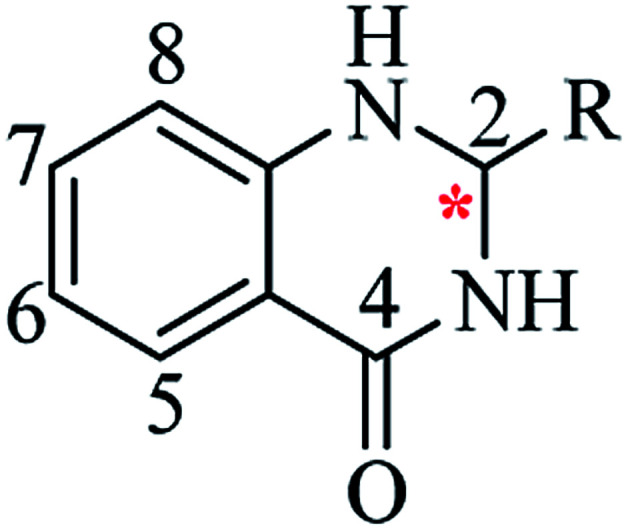
2,3-Dihydroquinazolin-4(1*H*)-one framework.

Nitrogen-containing heterocyclic scaffolds are quite common fragments in drugs and biologically active compounds.^1,2^ The 2,3-dihydroquinazolin-4(1*H*)-one (DHQ) is an important nitrogen-containing motif consisting of a phenyl ring condensed with a six-membered ring with two nitrogen atoms on positions 1 and 3, and a keto group on carbon 4 (Fig. 1). Most of the DHQ derivatives are substituted on the carbon 2 chiral center. Due to their attractive properties, 2-substituted DHQs are becoming a prominent synthetic intermediate for organic chemists and various methodologies are reported in the literature for their preparation as racemic mixtures. Although some asymmetric strategies have been attempted, the aminal chiral center is sensitive to racemization, making it difficult to synthesize pure enantiomers.^3^ The aim of the present review is to discuss reported methods for the synthesis of DHQ derivatives, highlighting their evolution towards alternative approaches and enantioselective strategies, and to summarize their use as synthons in organic chemistry and their important biological activities.

### 2,3-Dihydroquinazolin-4(1*H*)-one: a privileged scaffold

**Fig. 2:**
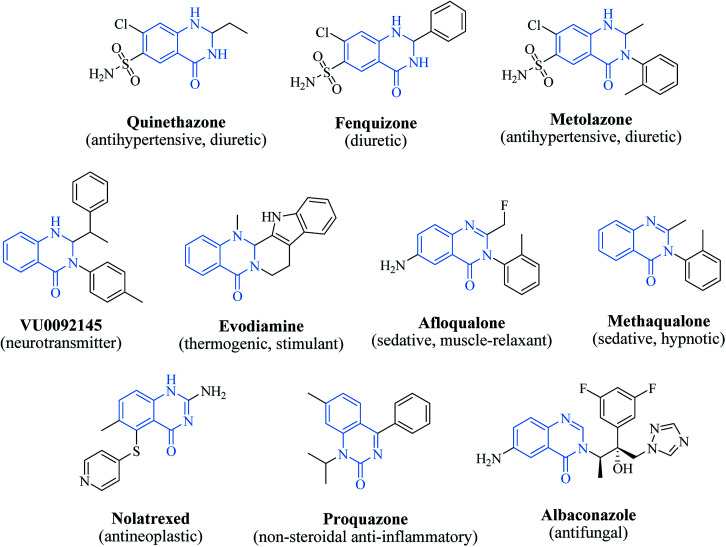
The “privileged scaffold” DHQ in marketed drugs.

**Fig. 3:**
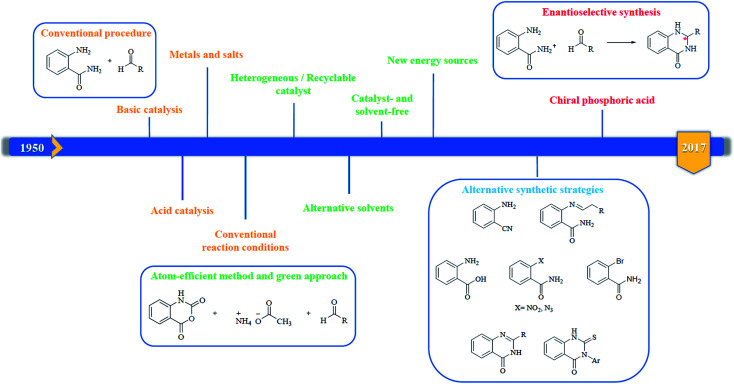
Evolution of the synthetic strategies to prepare DHQ core.

In 1988, Evans *et al.* introduced for the first time the concept of “privileged structures”. They are useful tools in the field of drug discovery since they represent suitable lead compounds for diverse receptors and the rational optimization of such structures could provide new receptor modulators and potential drugs.^4^ Medicinal chemists exploit the “privileged structures” to synthesize new libraries of compounds based on a central scaffold and screen them against various receptors implicated in different pathways, in some cases yielding biologically active compounds. In this regard, the DHQ core is emerging as a “privileged scaffold” and a variety of its derivatives, having diverse mechanism of action, are currently used for the treatment of various diseases.^5–11^ A panel of marketed drugs with the DHQ core is shown in Fig. 2. Besides these marketed drugs, a number of new DHQ derivatives have been designed that exhibit a wide range of pharmacological properties. Because of their importance, the synthesis of substituted DHQ derivatives has attracted much attention and different synthetic strategies have been developed. Since the classical protocols involved the use of toxic reagents and solvents in harsh reaction conditions, the evolution towards simple, clean, environmentally benign and high-yielding methods is gaining momentum (Fig. 3).

### Conventional procedure: cyclocondensation of anthranilamide and an aldehyde

**Scheme 1:**
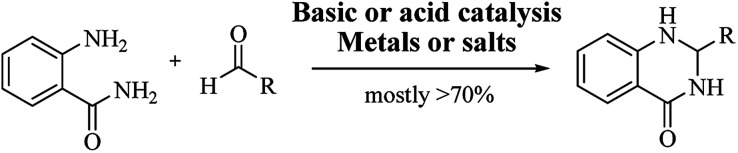
Direct cyclocondensation of anthranilamide and aldehyde under conventional conditions.

**Scheme 2:**
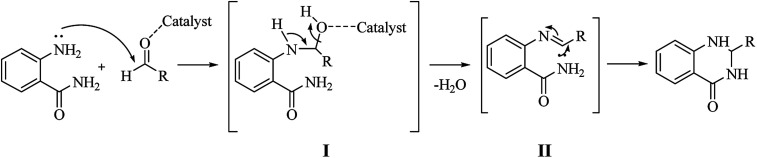
Presumed mechanism of the cyclocondensation of anthranilamide and an aldehyde.

Although numerous strategies have been developed for the construction of the DHQ core, the most common and simple synthetic route for the preparation of DHQs is the direct cyclocondensation of anthranilamide and an aldehyde (Scheme 1). In the past decades, different catalysts and organic solvents have been used to speed up and improve the general yield of the reaction (Table 1). Regardless of the used catalyst and/or solvent, the most presumed mechanism of cyclocondensation is shown in Scheme 2.^12^ The first step involves the nucleophilic attack of the nitrogen of the amino group of the anthranilamide on the carbonyl carbon of the aldehyde, promoted by the catalyst, resulting in the formation of hydroxyl intermediate I. Next, the catalyst promotes the formation of the Schiff base (II) from I through the removal of a water molecule. Finally, the imine undergoes intramolecular cyclization by nucleophilic attack of the nitrogen of the amide group on the imine carbon, to furnish the corresponding DHQ derivative.

**Table 1:** Conventional conditions for the cyclocondensation of anthranilamide and an aldehyde

Nature catalyst	Catalyst	Conditions	Time	Yield	Ref.
Strong base	NaOH	EtOH, reflux	1 h	60–86%	13–15
	NaOEt	EtOH, reflux	3–4 h	20–85%	14
Strong BrØnsted acid	HCl	EtOH, reflux	4 h	10%	14
	Conc. HNO_3_/HCl	Reflux → rt	5 → 30 min	>98%	16
	H_2_SO_4_	Solvent-free, MWI	Few min	68–78%	17
Sulfonic acid functionality	PTSA	Chlorobenzene, reflux	1 h	74%	13
		Benzene, reflux	4 h	70%	18
		DMAC, rt/reflux	1–2 h	75–95%	19 and 20
		EtOH, reflux	1 h	70–90%	21
	MES	Aq. EtOH (50%), MWI	5–20 min	83–96%	22
	Sulfanilic acid	Aq. EtOH (50%), 70 °C			23
	NaHSO_4_	EtOH, rt	0.5–5.5 h	91–97%	24
		H_2_O, grinding rt → 60 °C	0.5–7 h	54–97%	25
	SOCl_2_	EtOH, rt	30–35 min	93–95%	26
Weak BrØnsted acid	Formic acid	20 °C			12
	Malonic acid	Aq. EtOH (50%), rt	5–37 min	81–98%	27
Weak Lewis acid	H_3_BO_3_	Solvent-free, 120 °C	5 min	82–90%	28
Organic	T3P®	AcCN, rt	10–15 min	85–94%	29
Lewis acid	I_2_	ILs, 50 °C/80 °C	0.5–10 h	76–99%	30 and 31
		EtOAc, *hv*	1–15 h	66–93%	32
Lugol's solution	I_2_/KI	H_2_O, rt	2–12 h	47–95%	33
Organic	C_3_Cl_3_N_3_	AcCN, rt	8–20 min	60–96%	40
Lewis acid	Mn(CH_3_COO)_2_	EtOH, reflux	5 h		34
	ZrCl_2_	EtOH, rt	9–60 min	80–97%	35
	HgCl_2_	EtOH, 60 °C	1–2 h	88–94%	36
	Cp_2_TiCl_2_	EtOH, rt	7–9 min	95–98%	37
	InBr_3_	AcCN, rt	10–60 min	75–98%	38
	BiBr_3_	AcCN, rt	30 min	80–95%	39
	Sc(OTf)_3_	EtOH, 70 °C	20–40 min	85–92%	42
		Dry DCM, rt	4–7 h	85–94%	43
		PEG-400, 80 °C	2 h	78–90%	44
	Yb(OTf)_3_	EtOH, 80 °C	2–6 h	>95%	45
		IL, rt	6–8 h	85–96%	46
	Y(OTf)_3_	EtOH, rt	1.5 h	88–99%	47
Ammonium salt	NH_4_Cl	EtOH, rt/reflux	5–120 min	78–98%	48 and 49
	CAN	H_2_O, rt → 60 °C	1–8 h	62–97%	50
	TBAHS	MeOH, reflux	2 h	64–90%	51

**Scheme 3:**
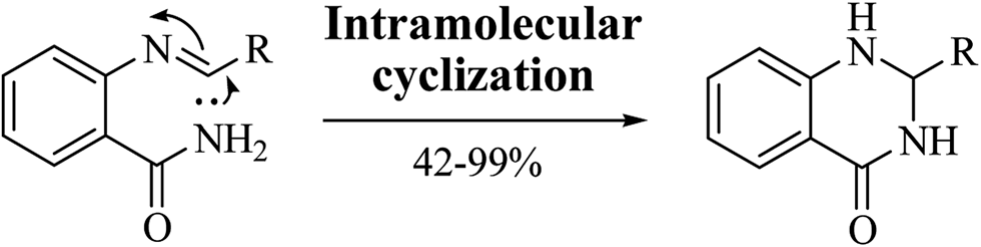
Intramolecular cyclization of a Schiff base

A variation of the traditional cyclocondensation of anthranilamide and an aldehyde is represented by the intramolecular cyclization of a Schiff base. As shown in Scheme 3, the nucleophilic attack of the nitrogen of the amide group on the imine carbon leads to the cyclic DHQ derivative.

### Basic catalysis

The base-catalyzed cyclocondensation of anthranilamide and an aldehyde was the first proposed strategy for the synthesis of DHQ derivatives. In 1967, Yale *et al.* prepared a new class of DHQs in the presence of 20% aqueous NaOH in refluxing EtOH. These compounds were tested for their inhibition of cell proliferation.^13^ Later, Bonola *et al.* used NaOH or NaOEt in absolute EtOH to prepare DHQs with antibacterial and antifungal activities.^14^ Ericsson *et al.* patented DHQ derivatives as anti-fertility agents; these compounds were prepared from anthranilamide and 1-naphthaldehyde in the presence of NaOH in refluxing absolute EtOH.^15^ The NaOH-catalyzed reactions gave moderate to high yields (60–85%) in 1 h compared to NaOEt, which took 3–4 h to complete the formation of DHQs (Table 1). Depending on the available aldehyde, the use of a strong base as catalyst was not always successful. Because of this limitation, different catalysts were commonly used for the synthesis of DHQs starting from anthranilamide and an aldehyde.

### Acid catalysis

As shown in Table 1, more frequently, the cyclocondensation of anthranilamide and an aldehyde is performed in acid catalysis. HCl in EtOH^14^ or in association with concentrated HNO_3_ (ref. 16) was used as catalyst under reflux conditions. As reported, DHQ derivatives were often synthesized in the presence of a catalytic amount of H_2_SO_4_ (ref. 17) or by using catalysts bearing sulfonic acid functionality. Among them, *p*-toluenesulfonic acid was used in different solvents, including boiling chlorobenzene,^13^ benzene,^18^*N*,*N*-dimethylacetamide^19,20^ and EtOH.^21^ 2-(*N*-Morpholino)ethanesulfonic acid, commonly used as a buffering agent in biology, also stood out as a mild acid catalyst for the cyclocondensation of anthranilamide and an aldehyde.^22^ The use of sulfanilic acid,^23^ NaHSO_4_,^24,25^ and SOCl_2_,^26^ as a catalyst in EtOH has also been described. Moreover, formic acid^12^ as catalyst and solvent, as well as malonic acid^27^ in aqueous EtOH (50%), were used for the synthesis of DHQs. Furthermore, boric acid (H_3_BO_3_),^28^ and propylphosphonic anhydride,^29^ usually employed in the Fisher indole synthesis and the Pictet–Spengler reaction, efficiently catalyzed the cyclocondensation of anthranilamide and aldehydes.

### Iodine and metal salts as catalysts

Molecular iodine (I_2_) emerged as a versatile, inexpensive and non-toxic catalyst, which serves as a Lewis acid. It is suitable in the cyclocondensation of anthranilamide and aldehydes, in ionic liquids,^30,31^ or in EtOAc.^32^ A synthesis in aqueous medium was also attempted. Due to poor solubility of I_2_ in water, 1 mol% of I_2_ as Lugol's solution (I_2_/KI) was used to prepare DHQs.^33^ In the presence of I_2_ as catalyst, DHQ derivatives were obtained with 66–95% yield, but the reactions required up to 15 h to complete.

Transition metal salts are excellent catalysts due to their kinetic stability, low toxicity and intrinsic metallic Lewis acidity. They were used in various organic transformations, including the cyclocondensation of anthranilamide and aldehydes.^34,40^ In particular, the use of transition metal salts reduced reaction time from hours to minutes without decreasing the yield. Compared to conventional Lewis acids, metal triflates are better catalysts in organic synthesis because of their chemical and physical properties, such as moisture and air-stability, recoverability, operational simplicity, and a strong tolerance to oxygen, nitrogen, phosphorus, and sulfur-containing reaction substrates and functional groups.^41^ Among them, scandium,^42–44^ ytterbium,^45,46^ and yttrium^47^ triflates were extensively used in various conditions to catalyze the cyclocondensation of anthranilamide and aldehydes, leading to DHQs with high yields (78–99%) in 0.3–8 h.

As shown in Table 1, even ammonium salts stood out as good catalysts for the preparation of DHQ derivatives.^48–51^ The reactions required short time and yields were up to 60%.

### Intramolecular cyclization of a Schiff base

The synthesis of DHQs through intramolecular cyclization of a Schiff base was performed under different conditions, providing DHQs with 42–99% yield in 1–16 h (Table 2). While strong basic conditions gave the final product in long time,^52^ the use of molecular nitrogen shortened the completion of the reaction.^53^ A rapid synthesis was obtained using metal oxide nanoparticles,^54^ and under catalyst-free conditions.^55,56^

**Table 2:** Reaction conditions for the intramolecular cyclization of a Schiff base

Substrate	Conditions	Time	% Yield	Ref.
Schiff base	NaH, THF, 0 °C → rt	16 h	42–91	52
	N_2_, EtOH, reflux	6 h	83	53
	Fe_3_O_4_ NPs, EtOH, reflux	1–3.5 h	94–98	54
	AcOH, reflux	1.5 h	80–92	55 and 56

### An atom-efficient method: one-pot three-component synthesis

**Scheme 4:**
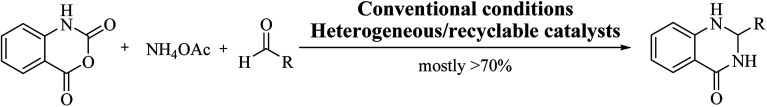
Preparation of DHQ through one-pot three-component reaction.

Although the cyclocondensation of anthranilamide and aldehydes is a facile and simple approach to obtain the DHQ core, more advantageous strategies to synthesize DHQ derivatives remain a desired goal in organic chemistry. The first evolution towards a more convenient approach was moving from a two-component reaction (anthranilamide and an aldehyde) to a one-pot three-component reaction (isatoic anhydride, ammonium acetate and an aldehyde) (Scheme 4). Initially, Staiger *et al.* suggested this strategy about half a century ago,^57^ and recently the advantages of multi-component reactions have been highlighted. Compared to conventional synthesis, the one-pot three-component reaction represents an attractive and atom-efficient method to efficiently prepare the DHQ framework.

**Scheme 5:**
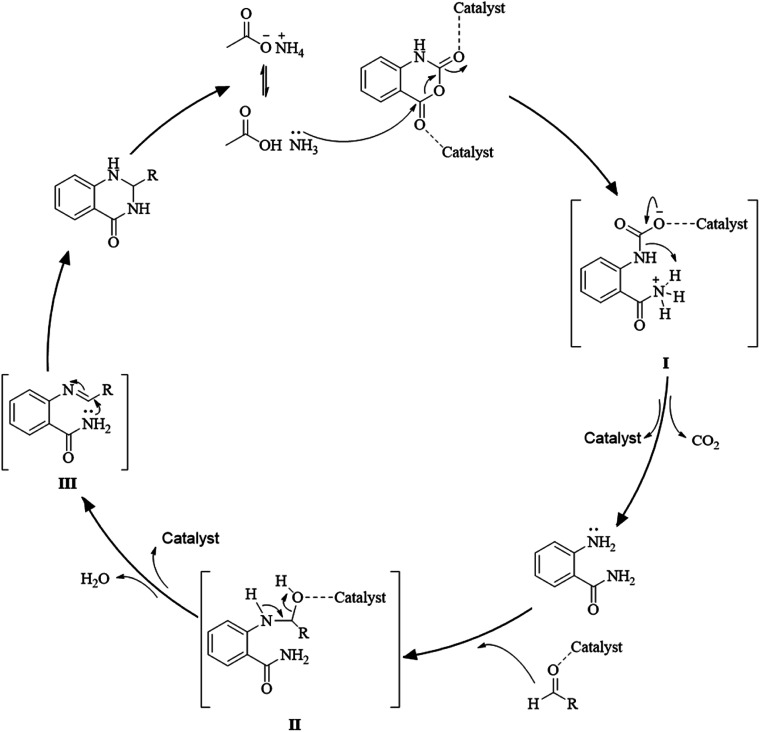
Plausible mechanism of the one-pot three-component synthesis of DHQ.

Multi-component reactions are characterized by (i) atom economy, incorporating all materials used in the process into the final product; (ii) high levels of diversity achieved simply by varying the reaction components; (iii) time-efficiency, since products are formed in a single step, allowing a fast probe of a chemical hypothesis; and (iv) simple experimental procedures, ideally there is no need to isolate the intermediates. Obviously, the adoption of such a strategy minimizes both waste production and cost.^58^ The plausible mechanism of the one-pot three-component synthesis of DHQs is shown in Scheme 5. Initially, the catalyst facilitates the nucleophilic attack of NH_4_OAc on the carbonyl carbon of the isatoic anhydride. Nucleophilic addition of ammonium leads to intermediate I while the following decarboxylation produces anthranilamide (II). Then, the reaction proceeds similarly to the cyclocondensation. The catalyst promotes the nucleophilic attack of the amino group of II on the carbonyl carbon of the aldehyde, resulting in the formation of the Schiff base (III) by removing a water molecule. Finally, the imine intramolecularly cyclizes by nucleophilic attack of the nitrogen of the amide group on the imine carbon, to furnish the corresponding DHQ derivative. Examples of various catalysts and organic solvents used in the reaction of isatoic anhydride, NH_4_OAc and an aldehyde are listed in Table 3.

**Table 3:** Reaction conditions for the one-pot three-component synthesis of DHQs

Nature Catalyst	Catalyst	Conditions	Time	% Yield	Ref.
BrØnsted acid	EDDA	H_2_O, reflux	7–10 h	86–93	59
	PFPAT	Toluene, reflux	3 h	88–95	60
Sulfonic acid functionality	PTSA	EtOH or H_2_O, reflux	3–12 h	50–71	61
	DBSA	H_2_O, USI	1–2 h	80–91	62
	Co(*m*-NBS)_2_	EtOH/H_2_O, reflux	0.5–4 h	82–98	65
	TBBDA or PBBS	EtOH/H_2_O, reflux	1–3.6 h	60–95	67
Sulfonic acid functionality/recyclable	Ce(SO_4_)_2_·4H_2_O	Solvent-free, 120 °C	30–50 min	85–97	63
	Al(MS)_3_·4H_2_O	EtOH/H_2_O, reflux	0.5–6.5 h	60–96	64
	Cu[C_6_H_5_SO_3_]_2_·6H_2_O	EtOH/H_2_O, reflux	0.5–6 h	71–95	66
	I_2_	EtOH or H_2_O, reflux	0.5–10 h	56–95	68
		Solvent-free, 115 °C	4–25 min	94–98	69
Lewis acid	SrCl_2_·6H_2_O	EtOH/H_2_O, reflux	0.5–6 h	42–94	70
	Cu(OTf)_2_	Toluene, reflux	12–18 h	50–85	72
	Yb(OTf)_3_	DMSO, 90 °C	15 h	36–41	73
Lewis acid/recyclable	Ga(OTf)_3_	EtOH, 70 °C	35–60 min	71–91	71
Recyclable	β-CD	H_2_O, 60 °C/reflux	1.5–5 h	78–92	76–78
	β-CD-SO_3_H	H_2_O, rt → 50 °C	25 min	80–97	79
	Starch solution	EtOH, 70 °C	4–8 h	73–94	80
	Starch sulfate	Solvent-free, 100 °C	5–55 min	75–96	81
	KAl(SO_4_)_2_·12H_2_O	EtOH, reflux	4–6 h	70–83	82
	Citric acid	H_2_O, 80 °C	1–7 h	50–94	83
	VB_1_	EtOH, reflux	2–6 h	75–94	84
	Amberlyst-15	Solvent-free, MWI	3–7 min	69–87	85
Heterogeneous/recyclable	Fe_3_O_4_ NPs	H_2_O, reflux	1.5–6 h	51–88	88
	Al/Al_2_O_3_ NPs	Solvent-free, 115 °C	8–30 min	65–98	89
	CuO NPs	EtOH/H_2_O, reflux or USI	10–30 min	73–95	90
	In_2_O_3_ NPs	H_2_O, 80 °C	4 h	78–88	91
	AIN NPs	Drop of H_2_O, 130 °C	3–7 h	62–73	92
	HAP NPs	H_2_O, 110 °C	0.7–2 h	80–90	93
	SPNP	H_2_O, reflux	1–6 h	79–97	95 and 96
	SiO_2_–FeCl_3_	Solvent-free, 80 °C	9 min to 2 h	45–91	97
	Fe_3_O_4_-SBA-15	EtOH, reflux	1.5–4 h	65–78	98
	Titanium-SiO_2_	H_2_O, 100 °C	2–8 h	86–95	99
Solid acidic Catalyst	SSA	EtOH, reflux	3–7 h	73–92	100
		H_2_O, 80 °C or solvent-free	3–6 h	70–86	101
	SBSSA	EtOH, 80 °C	0.5–4 h	75–90	102
	SBNPSA	EtOH, reflux	2.5–3 h	81–88	103
	LPCAHS-SiO_2_	H_2_O, 80 °C	1.5–4 h	72–95	104
	MCM-41-SO_3_H	Solvent-free, 115 °C	4–20 min	75–98	105
	SPC	Solvent-free, 70 °C	2.5–3.5 h	78–86	106
	H_3_BO_3_-MCM-41	Solvent-free, 80 °C	0.3–1 h	76–94	107
	Cellulose-H_3_BO_3_	Solvent-free, rt	3–40 min	79–92	108
	Al(H_2_PO_4_)_3_	Solvent-free, 100 °C	9–17 min	80–93	109
	H_3_PO_4_–Al_2_O_3_	Solvent-free, 100 °C	4min to 3 h	70–90	110
	PTA-DEAEC	EtOH, reflux	5–7 h	60–91	111
	Copolymer-PTSA	EtOH, reflux	5–7 h	70–94	112
	Montmorillonite K-10	EtOH, reflux	0.5–7 h	70–95	113,114
Heterogeneous/recyclable	Cu-CNTs	Solvent-free, MWI	5–23 min	87–99	116
	Co-CNTs	Solvent-free	5–20 min	85–96	117
	Co-MWCNTs	EtOH, USI	6–20 min	76–97	118
	Pt-MWCNTs	EtOH, USI	8–20 min	88–96	119
	732-resin	EtOH/H_2_O, 90 °C	0.5–5 h	85–95	122
	La^3+^/4 Å	AcCN, reflux	24 h	45–95	123
Acid-surfactant-Combined	Zn(PFO)_2_	EtOH/H_2_O, reflux	6–7 h	77–86	120
	TA-SDS	EtOH/H_2_O, grinding, rt	1 min to 24 h	87–93	121

### Conventional reaction conditions

In general, the one-pot three-component syntheses of DHQs were performed under acid-catalyzed conditions (Table 3). BrØnsted acid catalysts, such as ethylenediamine diacetate^59^ and pentafluorophenylammonium triflate^60^ were used under reflux conditions, giving DHQ derivatives with 86–95% yield in about 10 h or less. Catalysts bearing sulfonic acid functionalities were also widely used in the multi-component reactions.^61–66^ They significantly reduced the reaction time, generating DHQs with 50–98% yield. Recently, the use of *N*-halo sulfonamides as catalysts, broadly reported in organic synthesis, has been attempted in the one-pot three-component reaction.^67^ Metal-catalyzed multi-component synthesis of DHQ derivatives was also reported.^68–73^ Generally, the use of metals or Lewis acids as catalysts did not lead to great yields (<90%), except when I_2_ is used under solvent-free conditions at 115 °C^69^ (Table 3).

### Recyclable catalysts

To improve the one-pot three-component reaction using green chemistry, the use of recyclable catalysts was introduced to further minimize waste production (Table 3). Some Lewis acid catalysts had the advantage of being easily recovered after the reaction and recycled several times without considerable loss of reactivity.^63,64,66,71^

β-Cyclodextrin (β-CD) is a cyclic oligosaccharide, composed of seven glucose units connected “head-to-tail” by 1,4-links. This cyclic heptamer has a truncated cone shape, with a hydrophobic cavity in the center. Thus, β-CD is used in numerous applications in drug formulation. It is well known that hydrophobic and van der Waals interactions are involved in the inclusion of complex formation between guest molecules and β-CD.^74^ Due to its hydrophobic cavity, β-CD is widely used as a catalyst for a variety of organic reactions, providing a microenvironment whereby it catalyzes reactions through the formation of non-covalent interactions.^75^ β-CD is a suitable catalyst in the one-pot three-component synthesis of various DHQs producing 78–92% yield in aqueous media.^76–78^ DHQs were also obtained in less than half an hour in the presence of an inexpensive, safe and recyclable sulfonic acid-functionalized β-CD (β-CD-SO_3_H) as an efficient catalyst in green media.^79^

Starch is a renewable, biodegradable, and relatively inexpensive material. A starch aqueous solution in EtOH was employed as safe, non-toxic and reusable catalyst for the preparation of DHQ derivatives with 73–94% yields.^80^ Starch sulfate also reduced the reaction time to less than 1 hour.^81^

Furthermore, alum (KAl(SO_4_)_2_·12H_2_O),^82^ citric acid,^83^ and thiamine hydrochloride (VB_1_),^84^ catalyzed the one-pot three-component reaction in aqueous media. The protocols were longer (up to 7 h) but reported to be environmentally safe.

Amberlyst-15 is a cationic exchange resin. These types of resins, especially the macroporous ones, are recyclable catalysts for various organic syntheses, including the preparation of DHQs. Amberlyst-15 presents several advantages over conventional catalyst with respect to corrosion, product recovery, and selectivity.^85^

### Heterogeneous and reusable catalysts

Recently, the development of heterogeneous organic reactions has been gaining popularity. They are characterized by ease of handling, separation, recycling, and environmentally safe disposal.^86^ In the last decade, the field of nanoscience and nanotechnology has had tremendous growth. Nanoparticles (NPs) are defined as materials having 1–50 nm diameter, a size range where metals can show size-dependent properties. Because of their interesting structures and high catalytic activities, due to the wide surface/volume ratio that provides many active sites per unit area, NPs and particularly magnetic particles have emerged as useful heterogeneous catalysts in terms of selectivity, reactivity, and improved yields of products. In addition, the magnetic properties of NPs make complete recovery of the catalyst possible by means of an external magnetic field.^87^ As reported in Table 3, some metal and metal oxide NPs, exhibiting high surface/volume ratio, quantum size and quantum tunnel effects, efficiently catalyzed the one-pot three-component synthesis of DHQs, in 0.5–6 h.^88–91^ Nano-sized aluminum nitride, a non-toxic, low-cost and highly pure powder, was used as a solid source of ammonia in the multi-component synthesis of DHQs.^92^ Hydroxyapatite (HAP) NPs show ion-exchange ability, adsorption capacity, and acid–base properties. Because of their higher surface areas and lower particle size, HAP NPs provide greater catalytic activity in the synthesis of DHQs.^93^ Although NPs have advantages such as simplified isolation of the product, easy recyclability and recovery of the catalyst, the naked NPs could aggregate into large clusters, limiting their use. This problem could be solved by immobilizing the NPs on mesoporous substrates characterized by large surface area, high chemical and thermal stability and good compatibility, such as silica, polymers and carbon.^94^ The integration of mesoporous silica with magnetic NPs is certainly of great interest for practical applications. As shown in Table 3, different silica-supported NPs were used as catalysts for the one-pot three-component reaction, leading to 45–97% yield in 1–8 h.^95–99^ The use of solid acids, as non-toxic, low-cost and reusable catalysts, has also emerged in the synthesis of DHQs (Table 3). Among them, several acids bearing sulfonic moiety supported on silica catalyzed the multi-component synthesis under different conditions, providing DHQs with >70% yield in 0.5–7 h.^100–104^ MCM-41 is another type of ordered mesoporous silica material. Various acid-functionalized MCM-41,^105–107^ cellulose,^108^ and alumina,^109,110^ were used as solid recyclable acidic catalysts for the one-pot three-component synthesis of DHQs, under solvent-free conditions, affording 70–98% yield in less than 1 hour. On the other hand, solid acids used in refluxing EtOH lengthened the reaction time to 5–7 hours.^111,112^ Montmorillonite K-10 is one of the most important smectites, a phyllosilicate mineral species, used as a catalyst in organic synthesis. It is a clay with both BrØnsted and Lewis acid sites, with a high cation-exchange capacity. Montmorillonite K-10 is considered a solid acid that acts as heterogeneous catalyst for diverse syntheses, including the one-pot three-component cyclocondensation, and can be easily removed from the reaction mixture.^113,114^ Other interesting solid supports used in heterogeneous catalysis are carbon nanotubes (CNTs), due to the porosity, inertness, and low interactions between catalyst and support and good mechanical strength.^115^ The deposition of metal NPs on the external surface of CNTs and multi-walled CNTs (MWCNTs) are attractive for catalysis, since they increase the reactive surface area. As reported in Table 3, CNTs supporting transition metals efficiently catalyzed the one-pot three-component synthesis of DHQs, obtained with 76–99% yield in less than half an hour.^116–119^ About the same yield was obtained using acid-surfactant-combined catalysts.^120,121^ A strong acidic cation-exchange resin (732-resin),^122^ and a 4 Å molecular sieve modified with lanthanum(iii) (La^3+^/4 Å),^123^ were employed as heterogeneous and recyclable catalysts for the multi-component synthesis of DHQs.

### Greener and convenient approaches to obtain DHQ derivatives

In the new century, there has been an increasing demand for the development of sustainable chemistry. In 1998, Anastas and Warner published the “Twelve Principles of Green Chemistry”,^124^ whose main purpose was the pollution prevention. In this regard, green chemistry was committed to (i) decrease pollution-generating chemicals; (ii) limit the use of dangerous chemicals and exhaustible feedstock materials and scarce resources; and (iii) reduce the harmful effects of final products.^125–127^ The development of cleaner and safer chemical processes started with the use of heterogeneous and recyclable catalysts or alternative solvents, which are not volatile, flammable or toxic. Then, it moved to performing reactions under catalyst- and/or solvent-free conditions. Finally, it incorporated the replacement of conventional thermal equipment by non-conventional sources, such as microwave or ultrasound irradiations.

### Heterogeneous/recyclable catalysts

As shown in Table 4, recyclable catalysts, such as β-CD,^77^ amberlyst-15,^128^ and cerous methanesulfonate [Ce(MS)_3_],^129^ have been used for the cyclocondensation of anthranilamide and aldehydes. In general, the use of heterogeneous and reusable catalysts afforded high yields, up to 99%, in very short times. A number of solid acid catalysts, on various supports, such as silica,^130–138^ and clay,^139,140^ as well as different catalysts bearing sulfonic acid groups and anchored on cellulose,^141^ Wang resin,^142^ NPs,^143,144^ and other supports,^145,146^ were employed. Reusable acid-surfactant-combined catalysts,^147,148^ and heteropolyacids,^149–152^ have also emerged as good catalysts, due to their unique properties like high thermal stability, low cost, ease of preparation and recovery. They efficiently catalyze the synthesis of DHQs in aqueous media in 5–90 min.

**Table 4:** Catalysts for green chemistry approaches to synthesize various DHQs

Nature catalyst	Catalyst	Conditions	Time	% Yield	Ref.
Recyclable	β-CD	H_2_O, 60 °C	1.5 h	78–92	77
	Amberlyst-15	CH_3_CN, 80 °C	10–30 min	96–98	128
	Ce(MS)_3_	H_2_O, grinding technique	0.2–3 h	84–94	129
Solid acid	Sulfamic acid	H_2_O, 60 °C or MeOH, rt	15–180 min	57–95	130
	H_2_SO_4_–SiO_2_	rt	0.2–5 h	93–97	131
	PPA-SiO_2_	Solvent-free, rt	1.5–4 h	89–93	132 and 133
	Amberlyst-15 and SiO_2_–HClO_4_	CH_3_CN, 80 °C	45–120 min	55–90	134
	SiO_2_–ZnCl_2_	Solvent-free, 100 °C	6–80 min	51–95	135
	CAN·SiO_2_	CH_3_CN, rt	10–50 min	78–94	136
	Boehmite-SSA	EtOH, 80 °C	35–130 min	85–96	137
	Boehmite-Si-DSA	EtOH, reflux	30–190 min	92–98	138
	Montmorillonite KSF	CH_3_CN, rt	20–50 min	90–99	139
	HCNC-4	CH_3_CN, rt	15–30 min	82–99	140
	Cellulose-SO_3_H	CH_3_CN, rt	40–60 min	77–92	141
	Wang-OSO_3_H	H_2_O, 100 °C	0.4–1.1 h	78–88	142
	Fe_3_O_4_-SA-PPCA	EtOH, 80 °C	30–80 min	91–95	143
	MNPs-PSA	H_2_O, 70 °C	25–170 min	71–97	144
	SuSA	H_2_O, 70 °C	48–60 min	86–95	145
	SO_4_^2−^/ZrO_2_	EtOH, reflux	7–160 min	84–96	146
	Zr(DS)_4_	H_2_O, rt	8–45 min	83–97	147
	*p*-SAC	H_2_O, rt	18–90 min	64–94	148
	H_3_PW_12_O_40_	H_2_O, rt	8–10 min	79–97	149
	H_3_PW_12_O_40_	EtOH/H_2_O, 80 °C	12–18 h	71–94	140
	SiO_2_–H_3_PW_12_O_40_	EtOH, reflux	5–40 min	88–98	151
	Poly(VPyPS)-PW	EtOH, USI	6–16 min	74–96	152
Organocatalyst heterogeneous/recyclable	α-Chymotrypsin	EtOH, 60 °C	30–60 min	90–98	153
	Fe_3_O_4_–GO	EtOH, reflux	2.5–5 h	70–80	154
	GO nanosheets	H_2_O, rt	10–30 min	85–97	155
	Co-CNTs	Solvent-free, MWI	10–35 min	75–98	156
	Ag-CNTs	EtOH, USI	5–21 min	86–97	157
	CuCl_2_/Fe_3_O_4_-TEDETA	EtOH, 80 °C	25–100 min	94–98	158
	MCM-41-dtz-Ni	PEG-400, rt	10–35 min	90–98	159
	Fe_3_O_4_/TiCl_2_/cellulose	EtOH, rt	6–15 min	79–96	160
	Sc(OTf)_3_	PEG-400, 80 °C	2 h	78–90	161

Enzymes have received great attention as sustainable and biodegradable catalysts for the synthesis of biologically active compounds. Among them, α-chymotrypsin rapidly catalyzes the cyclocondensation of anthranilamide and aldehydes with 90–98% yield.^153^ Transition metal-based heterogeneous systems were efficiently used as recyclable catalysts for the cyclocondensation of anthranilamide and aldehydes, yielding > 70% DHQs.^154–161^

### Alternative solvents

The choice of the solvent for a desired chemical process can have profound economic and environmental consequences. For this reason, there has been significant interest in using alternative “clean” solvents, mostly aqueous media and ionic liquids. Water is readily available, cheap, non-toxic, non-flammable and is very attractive from both an economical and environmental point of view.^162^ The use of ionic liquids as reaction media and catalysts also gives a solution to solvent emission and catalyst-recycling problems. Ionic liquids present many important features, such as negligible vapor pressure, non-inflammability, immiscibility with non-polar solvents, reasonable thermal and chemical stability and recyclability.^163^

Various catalysts were used in aqueous media to synthesize DHQ derivatives, both from the cyclocondensation of anthranilamide and aldehydes, and the one-pot three-component reaction. A hydrotropic solution^164^ and a deep eutectic solvent^165^ were used under catalyst-free conditions (Table 5). Ionic liquids were also widely employed for the synthesis of DHQ derivatives, in different reaction conditions (Table 6). Imidazolium-based ionic liquids^30,31,46,166–171^ afforded DHQ derivatives with 70–99% yield, in 0.5–10 h unless used under microwave irradiation (MWI).^172^ The triazolium-based reactions take less than half an hour to complete the one-pot three-component reaction.^173^ Ionic liquids bearing sulfonic acid functionality were used under solvent-free conditions and rapidly catalyzed the synthesis of DHQs.^174,175^ Although basic ionic liquids^171–178^ and a glycerol based ionic liquid with a boron core^179^ were also used under solvent-free conditions, they catalyzed the reaction in a longer time (10–90 min).

**Table 5:** Alternative solvents: aqueous media for greener approaches to synthesize of DHQs

Nature solvent	Solvent	Conditions	Time	% Yield	Ref.
Aqueous medium	Aq. EtOH (50%)	MES, MWI	5–20 min	83–96	22
		Sulfanilic acid, 70 °C			23
		Malonic acid, rt	5–37 min	81–98	27
	EtOH/H_2_O	Al(MS)_3_·4H_2_O, reflux	0.5–6.5 h	60–96	64
		Co(*m*-NBS)_2_, reflux	0.5–4 h	82–98	65
		Cu[C_6_H_5_SO_3_]_2_·6H_2_O, reflux	0.5–6 h	71–95	66
		TBBDA or PBBS, reflux	1–3.6 h	60–95	67
		SrCl_2_·6H_2_O, reflux	0.5–6 h	42–94	70
		CuO NPs, reflux or USI	10–30 min	73–95	90
		Zn(PFO)_2_, reflux	6–7 h	77–86	120
		TA-SDS, grinding, rt	1 min-24 h	87–93	121
		732-resin, 90 °C	0.5–5 h	85–95	122
		H_3_PW_12_O_40_, 80 °C	12–18 h	71–94	150
	EtOH or H_2_O	PTSA, rflux	3–12 h	50–71	61
		I_2_, reflux	0.5–10 h	56–95	68
	H_2_O	NaHSO_4_, grinding	0.5–7 h	54–97	25
		I_2_/KI, rt	2–12 h	47–95	33
		CAN, rt → 60 °C	1–8 h	62–97	50
		EDDA, reflux	7–10 h	86–93	59
		DBSA, USI	1–2 h	80–91	62
		β-CD, 60 °C/reflux	1.5–5 h	78–92	76–78
		β-CD-SO_3_H, rt → 50 °C	25 min	80–97	79
		CA, 80 °C	1–7 h	50–94	83
		Magnetic Fe_3_O_4_ NPs, reflux	1.5–6 h	51–88	88
		In_2_O_3_ NPs, 80 °C	4 h	78–88	91
		HAP NPs, 110 °C	0.7–2 h	80–90	93
		SPNP, reflux	1–6 h	79–97	95 and 96
		Titanium-SiO_2_, 100 °C	2–8 h	86–95	99
		SSA, 80 °C	3–6 h	70–86	101
		*L*PCAHS- SiO_2_, 80 °C	1.5–4 h	72–95	104
		Ce(MS)_3_, grinding	0.2–3 h	84–94	129
		Sulfamic acid, 60 °C or MeOH, rt	15–180 min	57–95	130
		Wang-OSO_3_H, 100 °C	0.4–1.1 h	78–88	142
		MNPs-PSA, 70 °C	25–170 min	71–97	144
		SuSA, 70 °C	48–60 min	86–95	145
		Zr(DS)_4_, rt	8–45 min	83–97	147
		*p*-SAC, rt	18–90 min	64–94	148
		H_3_PW_12_O_40_, rt	8–10 min	79–97	149
		GO nanosheets, rt	10–30 min	85–97	155
Hydrotropic solution	NaPTS (50%)	Catalyst-free, 60 °C	50–95 min	78–95	164
Deep eutectic solvent	L-(+)-TA-DMU	Catalyst-free, 90 °C	4 h	79	165

**Table 6:** Alternative solvents: ionic liquids for green chemistry approaches to synthesize various DHQs

Ionic liquid	Structure	Conditions	Time	% Yield	Ref.
[bmim^+^][BF_4_^−^]	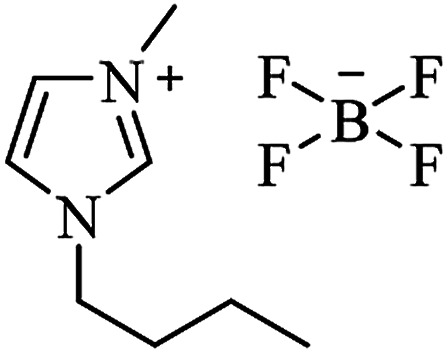	I_2_, 50 °C	4–10 h	76–98	30
		I_2_, 80 °C	0.5–1 h	90–99	31
		Catalyst-free, 70 °C	1.5–2.5 h	78–94	166
[bmim^+^][Br^−^]	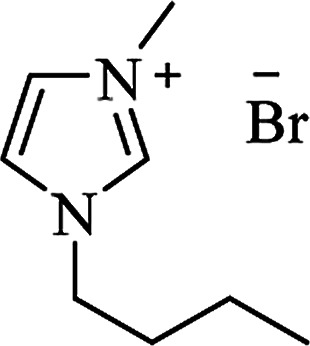	I_2_, 80 °C	7–10 h	82–91	167
		Yb(OTf)_3_, rt	6–8 h	85–96	46
		Catalyst-free, 80 °C			168
[bmim^+^][PF_6_^**−**^]	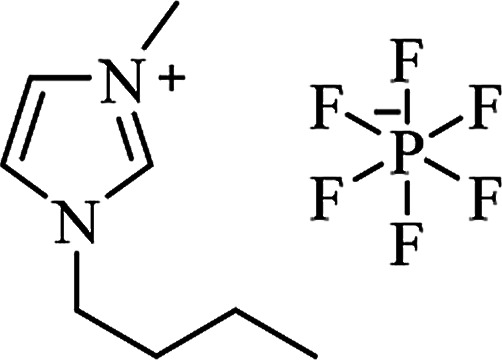	Solvent-free, 75 °C	35–75 min	77–94	169
[bmim^+^][HSO_4_^**−**^]	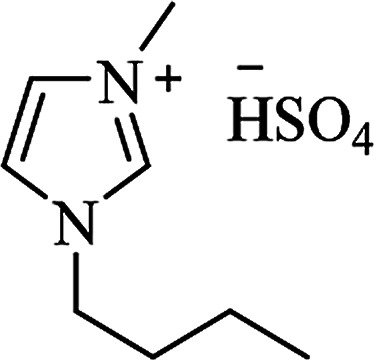	H_2_O, reflux	3–4 h	70–85	170
[msim^+^][HSO_4_^**−**^]	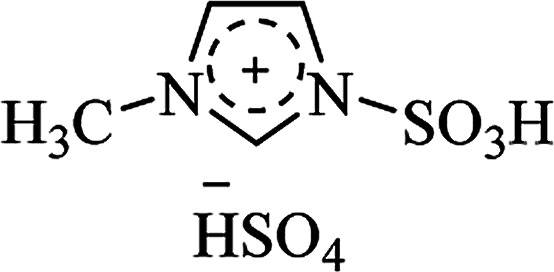	EtOH/H_2_O, reflux	25–45 min	80–95	171
[bdbim^+^][Br^**−**^]	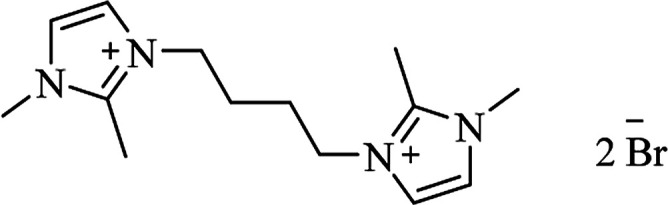	MWI, 100 °C	3–7 min	89–97	172
IPTT	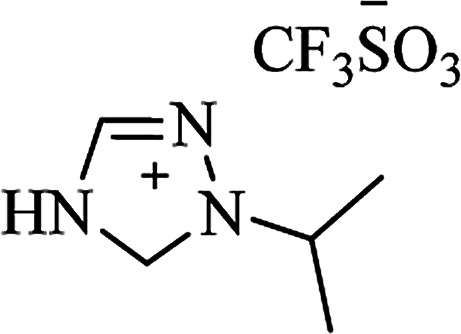	EtOH, 30 °C	1–24 min	10–96	173
[PY(CH_2_)_4_SO_3_H][HSO_4_]	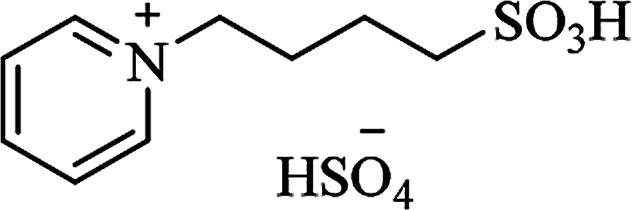	A300SiO_2_ solvent-free, 110 °C	10–14 min	81–90	174
[tpps^+^][TS^**−**^]	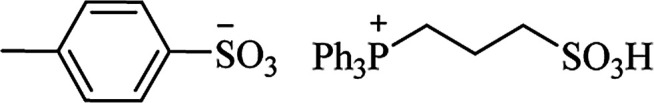	Solvent-free, 80 °C	4–30 min	85–96	175
TBAB	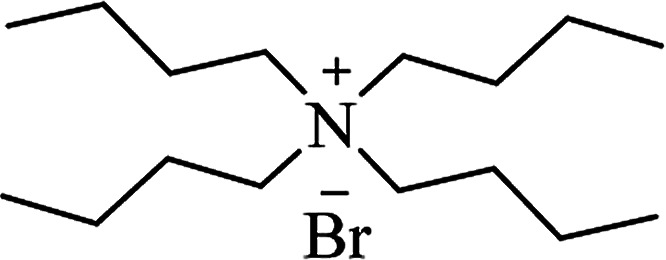	Solvent-free, 100 °C	45–90 min	72–84	176
		Solvent-free, 105 °C or H_2_O, 70 °C	25–75 min	67–91	177
Basic ionic liquid	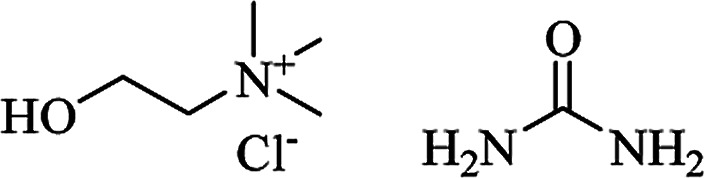	rt	3–5 h	80–90	178
H[Gly_2_B]	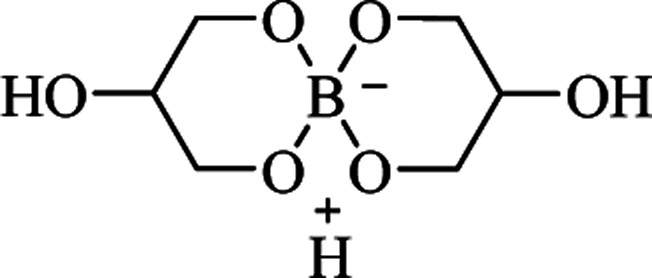	Solvent-free, 60 °C	10–55 min	83–92	179

### Catalyst- and solvent-free reactions

The challenge for a sustainable environment requires the development of greener and cleaner chemical processes that can avoid the use of harmful solvents and catalysts. In this sense, new strategies have been developed to synthesize DHQ derivatives in solvent- and/or catalyst-free conditions (Table 7). The cyclocondensation of anthranilamide and aldehydes was performed in the presence of sodium dihydrogen phosphate (NaH_2_PO_4_),^180^ acidic catalysts,^181–183^ and various heterogeneous systems, under solvent-free conditions, leading to various DHQs with 50–98% yield in a few minutes. On the contrary, a simple and environmentally benign procedure, without any catalyst, lengthened the reaction time to hours, although producing similar yield.^184–192^ The one-pot three-component reaction was also efficiently performed under solvent-free conditions in the presence of heterogeneous catalysts using conventional heating or MWI. The absence of catalyst again afforded 74–97% yield but in longer reaction times (1–6 h).^193–197^ Urea or thiourea was used in the multi-component synthesis of DHQs as the ammonia surrogate under catalyst-free conditions.^198^ A very efficient and fast synthesis of DHQs was attempted under both catalyst- and solvent-free conditions (>90% yield, in 3–10 min).^199,200^

**Table 7:** Catalyst- and/or solvent-free synthesis of DHQs

Reaction	Catalyst	Conditions	Time	% Yield	Ref.
Cyclocondensation	H_2_SO_4_	Solvent-free, MWI	Few min	68–78	17
	H_3_BO_3_	Solvent-free, 120 °C	5 min	82–90	28
	H_3_BO_3_-MCM-41	Solvent-free, 80 °C	20–65 min	76–94	107
	Cellulose-H_3_BO_3_	Solvent-free, rt	3–40 min	79–92	108
	Co-CNTs	Solvent-free, rt	5–20 min	85–96	117
	PPA-SiO_2_	Solvent-free, rt	1.5–4 h	89–93	132
	Co-CNTs	Solvent-free, MWI	10–35 min	75–98	156
	H_3_BO_3_ or NaH_2_PO_4_	Solvent-free, 120 °C	3–15 min	50–92	180
	Citric acid	Solvent-free, grinding	10–20 min	65–98	181
	Lemon juice	Solvent-free, rt	7–10 min	82–85	182
	Lactic acid	Solvent-free, 70 °C	0.3–6 h	80–92	183
	Catalyst-free	L-(+)-TA-DMU, 90 °C	4 h	79	165
		AcOH, rt	4 h	35–49	184
		Dry MeOH, reflux	5 h	55–73	185
		TFE, reflux	0.4–56 h	40–98	186
		DCM, reflux	2–3 d	98	187
		PEG-400, 100–110 °C	4–10 h	78–92	188
		Glycerol, 80 °C	1–4 min	87–95	189
		H_2_O, 90 °C	1–6 h	67–94	190 and 191
		H_2_O, reflux	0.5–27 h	73–99	192
One-pot Three-component	Ce(SO_4_)_2_·4H_2_O	Solvent-free, 120 °C	30–50 min	85–97	63
	I_2_	Solvent-free, 115 °C	4–25 min	94–98	69
	Starch sulfate	Solvent-free, 100 °C	5–55 min	75–96	81
	Amberlyst-15	Solvent-free, MWI	3–7 min	69–87	85
	Al/Al_2_O_3_ NPs	Solvent-free, 115 °C	8–30 min	65–98	89
	SiO_2_–FeCl_3_	Solvent-free, 80 °C	9–120 min	45–91	97
	SSA	Solvent-free, rt	3–6 h	70–86	101
	MCM-41-SO_3_H	Solvent-free, 115 °C	4–20 min	75–98	105
	SPC	Solvent-free, 70 °C	2.5–3.5 h	78–86	106
	Cellulose-H_3_BO_3_	Solvent-free, rt	3–40 min	79–92	108
	Al(H_2_PO_4_)_3_	Solvent-free, 100 °C	9–17 min	80–93	109
	H_3_PO_4_–Al_2_O_3_	Solvent-free, 100 °C	4–180 min	70–90	110
	Cu-CNTs	Solvent-free, MWI	5–23 min	87–99	116
	SiO_2_–ZnCl_2_	Solvent-free, 100 °C	6–80 min	51–95	135
	Catalyst-free	NaPTS (50%), 60 °C	3–5 h	80–90	178
		AcOH, reflux	1–2.5 h	79–97	193
		TFE, reflux	3 h	80–97	194
		[bmim^+^][PF_6_^**−**^], 75 °C	15–55 min	74–93	195
		PEG-400, 120–125 °C	1–6 h	80–97	196
		Glycerol, 80 °C	2 h	88–90	197
		EtOH, reflux	6 h	80–92	198
		Solvent-free, 70 °C	10 min	87–96	199
		Solvent-free, 120 °C or MWI	3 min	90–97	200

### New energy sources: microwave and ultrasound irradiations

MWI in organic synthesis is commonly used because it facilitates heat transfer better in chemical reactions. The efficiency of MWI heating results in a dramatic reduction in reaction times to minutes as compared to conventional heating methods taking several hours. From an economic and environmental viewpoint, the use of MWI provides unique chemical processes, characterized by enhanced reaction rates, sometimes higher yields, greater selectivity, and ease of manipulation.^201,202^ Previously, efforts were made to synthesize DHQs under MWI (Table 8). Among them, the cyclocondensation of anthranilamide, under acidic catalysis,^203^ as well as the one-pot three-component reaction, in the presence of l-proline in water.^204^ The ultrasound irradiation (USI)-assisted reactions have also become increasingly popular in organic synthesis. Due to faster reactions, MWI and USI allow the elimination or minimization of side products formation. They are frequently used in the pharmaceutical industry and may pave the way towards a greener and more sustainable approach to chemical synthesis.^205^ A variety of organic reactions were carried out within short times under USI, including the synthesis of DHQs, in the presence of amberlyst-15 (ref. 206) and in other different conditions (Table 8).

**Table 8:** New energy sources for greener approaches to synthesize DHQs

Reaction	Energy source	Catalyst	Conditions	Time	% Yield	Ref.
Cyclocondensation	MWI	H_2_SO_4_	Solvent-free	Few min	68–78	17
		MES	Aq. EtOH (50%), 600W	5–20 min	83–96	22
		Cu-CNTs	Solvent-free, 300W	5–23 min	87–99	116
		Catalyst-free	Solvent-free, 300W	3 min	90–97	200
		PTSA	AcOH, 300W	5–20 min	66–95	203
	USI	Poly(VPyPS)-PW	EtOH, rt	6–16 min	74–96	152
		Ag-CNTs	EtOH, 75 °C	5–21 min	86–97	157
		Amberlyst-15	CH_3_CN, rt	1–5 min	95–98	206
One-pot three-component	MWI	Amberlyst-15	Solvent-free, 360W	3–7 min	69–87	85
		Co-CNTs	Solvent-free, > 500W	10–35 min	75–98	156
		Catalyst-free	[bdbim^+^][Br^−^], 100 °C	45–90 min	72–84	172
		Catalyst-free	Solvent-free, 300W	1–6 h	80–97	200
		l-Proline	H_2_O, 100 °C, 250W	7–8 min	84–95	204
	USI	DBSA	H_2_O, 40–42 °C	1–2 h	80–91	62
		CuO NPs	EtOH/H_2_O, reflux	10–30 min	73–95	90
		Co-MWCNTs	EtOH, 35 kHz, 40 °C	6–20 min	76–97	118
		Pt-MWCNTs	EtOH, 60 °C	8–20 min	88–96	119

### Alternative synthetic strategies

Although the cyclocondensation of anthranilamide and the one-pot three-component reaction of isatoic anhydrides, ammonium acetates and aldehydes are the main ways to synthesize DHQ derivatives, other synthetic strategies have also been developed (Table 9).

**Table 9:** Reaction conditions for the alternative synthetic strategies

Starting material	Substrate	Conditions	Time	% Yield	Ref.
Anthranilamide	Benzyl	i: ZnCl_2_, AcOH, reflux	3 h	76	207
		ii: NaOH, EtOH, rt	1 h	88	
	2-Oxo(alkyl)acetate	i: PTSA, toluene, reflux	4–7 h	65–80	208
		ii: KOH, MeOH, rt	24 h	94	
	4′-Bromo acetophenone alcohol	I_2_, THF, 50 °C	6 h	86	209
		5mol% (PPh_3_)_3_Ru(CO)H_2,_ 5mol% xantphos, 2.5eq. crononitrile, 20mol% NH_4_Cl, toluene, N_2_, reflux	14 h	69–78	210
	*Gem*-dibromomethylarene	*t*-BuOK, Py/DMF, 80 °C	4–4.5 h	60–90	211
	Dicyanoepoxide	CH_3_CN, reflux	20 h	45–82	212
	Terminal alkynes	5mol% Ph_3_PauNTf_2_, toluene, 100 °C	12 h	60–97	213
	Alkynes	5mol% PtBr_2_ or Au(PPh_3_)Cl, MeOH, 80 °C	24 h	70–98	214
2-Amino-benzonitrile	Aldehyde	ZnCl_2_, DMF, reflux	1–24 h	47–88	215–217
		ChOH, H_2_O, 80 °C	0.5–2 h	82–96	218
		K_3_PO_4_, H_2_O, 100 °C	8 h	28–80	219
		1,3-Dipropylimidazole, solvent-free, rt	1–2 h	18–98	220
		i: 20% KOH, reflux	7 h	39–49	221
		ii: AcOH, rt	4 h		
		Amberlyst A26 OH, EtOH/H_2_O, 50–60 °C	2.5–4 h	75–93	222
*o*-Nitrobenzamide	Aldehyde	TiCl_4_/Zn, THF, reflux	2 h	79–91	223
		TiCl_4_/Sm, THF, reflux	2 h	71–92	224
		Fe, AcOH, 115 °C	30 min	73–94	225
		SnCl_2_·H_2_O, EtOH, rt	2–4 h	82–86	226
		SmI_2_, THF, rt	3–4 h	60–85	227
*o*-Nitrobenzamide *o*-Azidobenzamide	Aldehyde	SmI_2_, MeOH, reflux	20 h	69–89	228
*o*-Azidobenzamide	Aldehyde	SmI_2_, THF/MeOH, rt→reflux	2–4 h	69–88	229
2-Aminobenzoic acid	Amine	i: SiCl_4_, Py, rt	6–24 h	36–81	230
		ii: SMEAH, toluene, reflux	24 h		
		iii: ClCO_2_Et, Py, rt	48 h	46–88	
	Aldehyde	i: Triphosgene, dry THF, rt	30 min	72	231
		ii: 28% aq. NH_4_OH, THF, rt	2 h	94	
		iii: PTSA, MeOH, rt	2 h	84	
2-Halobenzamide	Aldehyde	CuBr, l-proline, DMSO, 100 °C	5 h	85	232
	Aniline	CuBr_2_, K_2_CO_3_, DMF, 130 °C	4 h	67	233
Quinazolinone	—	NaBH_4_, diglyme, 85 °C	1 h	50	234
	—	NaBH_4_, AcOH, 50 °C	48 h	50–52	235
	—	NaBH_4_CN, AcOH, rt	24 h	33	236
2-Thioxo-4(3*H*)QZ	—	NiCl_2_, NaBH_4_, dry MeOH, rt	0.5–24 h	79–92	237
2-Phenyl-ethyl-anthranilate	—	NH_4_OAc, AcOH, 50 °C → 90 °C	90–150 min	76–81	238
Benzylaniline	Anthranilamide	O_2_, AcOH, rt	24 h	62–83	239

### Cyclocondensation of anthranilamide and different substrates

**Scheme 6:**
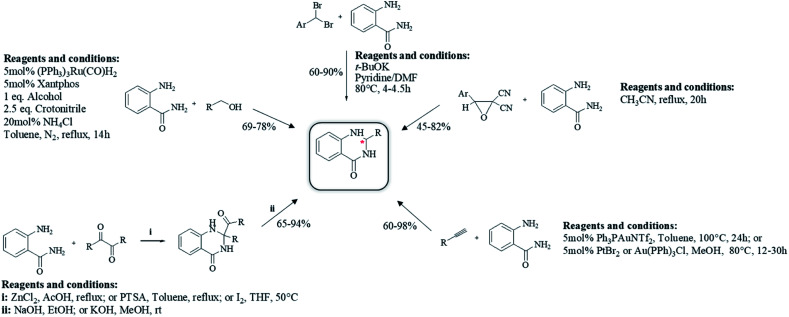
Alternative synthetic strategies of cyclocondensation of anthranilamide and different substrates.

Anthranilamide was the most common starting material for the preparation of DHQs. Other than aldehydes, other substrates were used for the cyclocondensation with anthranilamide in different conditions to give DHQ derivatives (Scheme 6).

First, the cyclocondensation of anthranilamide and oxo-compounds, such as benzil,^207^ 2-oxo(alkyl)acetates,^208^ and 4′-bromoacetophenone,^209^ were attempted to give DHQs in different conditions. The obtained 2,2-disubstituted DHQ was then cleaved in ethanolic/methanolic hydroxide to give the respective derivative. Alcohols were used in a ruthenium-catalyzed cyclocondensation with anthranilamide.^210^ Cyclocondensations of anthranilamide and *gem*-dibromomethylarenes, as aldehyde equivalents, were performed in the presence of potassium *tert-*butoxide (*t*-BuOK), in anhydrous pyridine and *N*,*N*-dimethylformamide (DMF).^211^ Anthranilamide also reacts with dicyanoepoxide to give DHQs in refluxing CH_3_CN.^212^

The direct hydroamination/hydroarylation and double hydroamination of alkynes, followed by the cyclocondensation with anthranilamide was also exploited for the synthesis of DHQ derivatives.^213,214^ These alternative strategies afforded DHQs with 45–97% yield in 1–24 h (Table 9).

### Reductive cyclocondensation of 2-aminobenzonitrile/*o*-nitrobenzamide/*o*-azidobenzamide and aldehydes

**Scheme 7:**
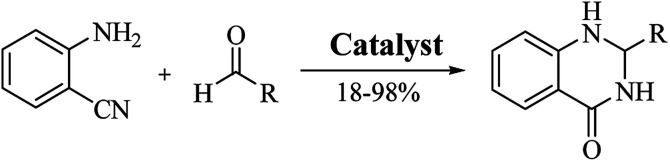
Cyclocondensation of 2-aminobenzonitrile and an aldehyde for the preparation of DHQs.

**Scheme 8:**
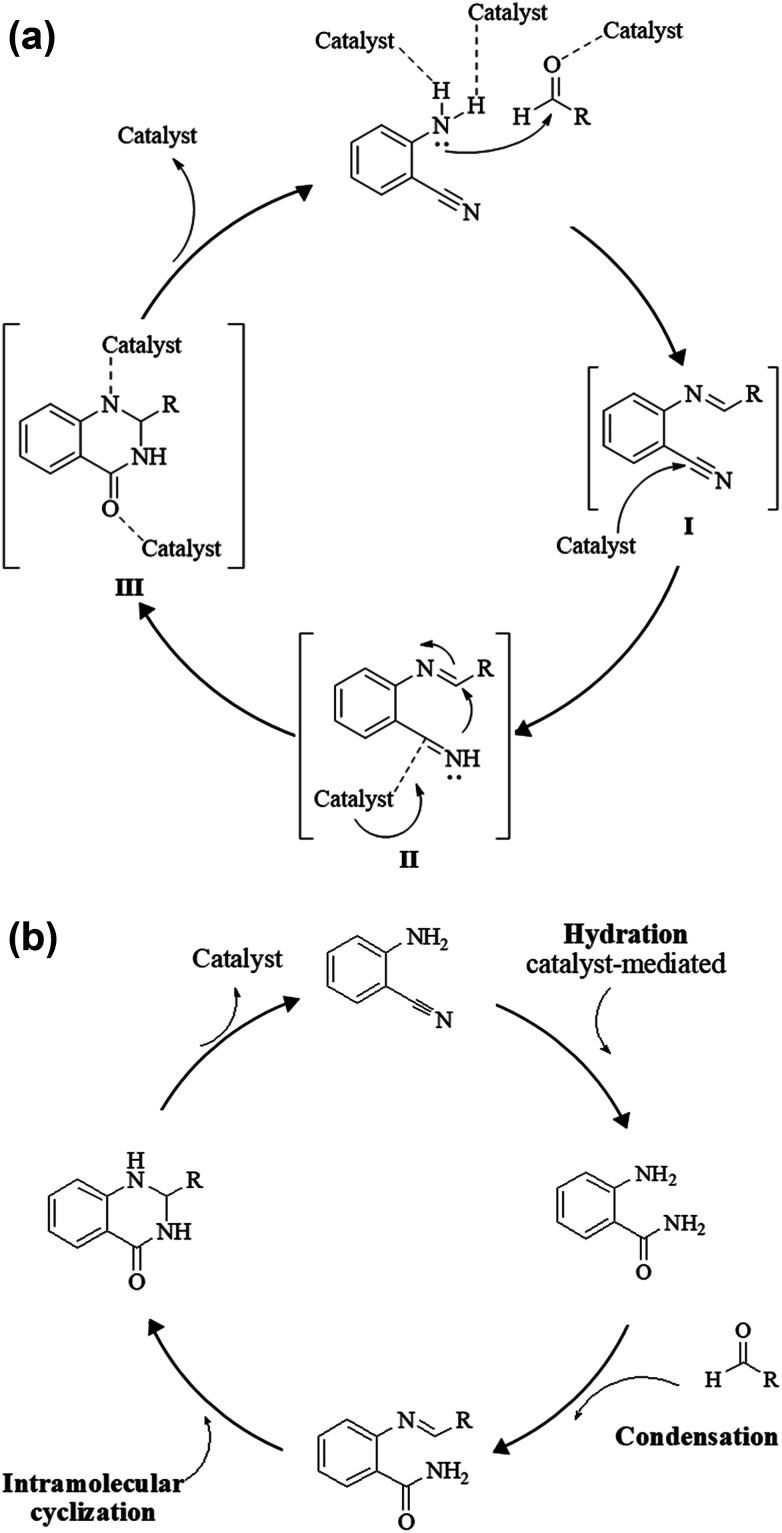
(a) Plausible mechanism of the cyclocondensation of 2-aminobenzonitrile and an aldehyde through formation of Schiff base. (b) Plausible mechanism of the cyclocondensation of 2-aminobenzonitrile and an aldehyde through hydration to anthranilamide.

An alternative method starting from 2-aminobenzonitrile and aldehydes was developed to synthesize DHQs (Scheme 7) and two plausible mechanisms have been proposed. The first was the formation of a Schiff base and subsequent cyclocondensation in the presence of various catalysts (Scheme 8a), yielding 18–98% DHQs in 0.5–8 h (Table 9).^215–220^ The second proposed mechanism was the hydration of the 2-aminobenzonitrile to anthranilamide in different conditions (Scheme 8b) by means of potassium hydroxide (KOH),^221^ or amberlyst A26 OH.^222^

**Scheme 9:**
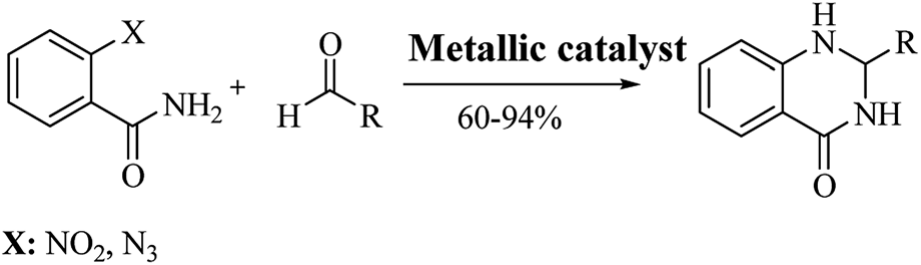
Reductive cyclocondensation of *o*-nitrobenzamide/*o*-azidobenzamide and an aldehyde.

The reductive cyclization of *o*-nitrobenzamide^223–227^ and/or *o*-azidobenzamide^228,229^ and aldehydes yield DHQ derivatives in the presence of metallic catalysts with 60–94% yield in short reaction times (Scheme 9; Table 9).

### Other strategies

**Scheme 10:**
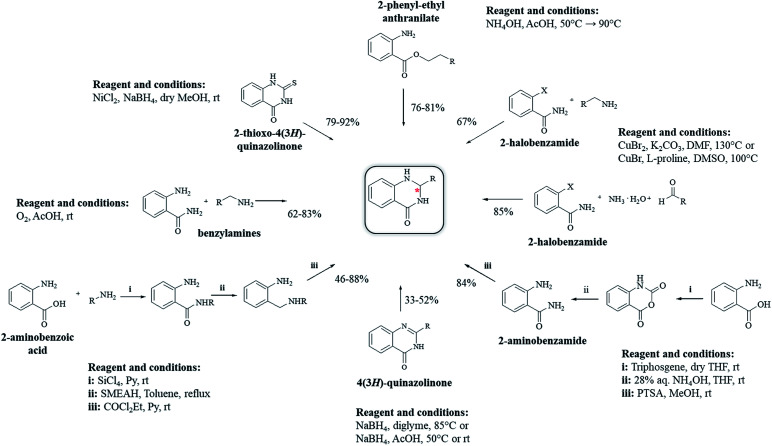
Alternative synthetic strategies from diverse starting materials.

Other strategies have been developed to synthesize DHQ derivatives starting from various substrates (Scheme 10) giving 39–94% yield in 0.5–48 h (Table 9). One such method employed 2-aminobenzoic acid as a starting material to prepare DHQ derivatives. The condensation of the 2-aminobenzoic acid with amines in the presence of SiCl_4_ led to the corresponding substituted anthranilamides, subsequently reduced with sodium bis(2-methoxyethoxy)-aluminum hydride to *o*-aminobenzylamines. Then, the latter compounds were cyclized into DHQs by means of ethylchloroformate/pyridine.^230^ Another strategy converted the 2-aminobenzoic acid in isatoic anhydride by means of triphosgene in dry THF, then converted to anthranilamide using 28% NH_4_OH solution. The cyclocondensation with the aldehyde in the presence of *p*-toluenesulfonic acid, in refluxing MeOH, gave the respective DHQ.^231^ The copper-catalyzed cyclocondensation of 2-halobenzamide and aldehyde in the presence of aqueous ammonia,^232^ and 2-halobenzamide with anilines,^233^ also gave DHQs derivatives. DHQs could also be prepared by reduction from 4(3*H*)-quinazolinones (QZs) with NaBH_4_,^234,235^ or NaBH_4_CN.^236^ DHQs were also obtained by reductive desulfurization of 2-thioxo-4(3*H*)-quinazolinones with nickel boride, using nickel(ii) chloride (NiCl_2_) and NaBH_4_.^237^ The intramolecular cyclization of 2-phenyl-ethyl anthranilate in the presence of NH_4_OAc led to the respective DHQs.^238^ The oxidation of benzylamines to the corresponding *N*-benzylbenzaldimines was also investigated and used for the synthesis of DHQ derivatives.^239^

### Enantioselective synthesis of DHQ derivatives

**Scheme 11:**
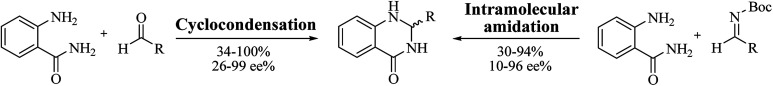
Enantioselective synthesis of DHQs.

Most of the reported methods allow the synthesis of DHQ derivatives as racemic mixtures. In certain examples, the (*S*)-enantiomer of DHQs had better antiproliferative activity, compared to the (*R*)-enantiomer, even though the racemic mixture showed a similar potency to the pure *S*-enantiomer.^3^ Chinigo *et al.* first proved that a (*S*)-enantiomer of DHQ binds to tubulin, showing antiproliferative activity in different cancer cell lines. They obtained the pure (S)-enantiomer using trifluoracetic acid in CH_3_CN with 34–86% yield and 79–91% enantiomeric excess percentage (ee%) in 1.5 h.^3^ Although the enantioselective synthesis of DHQs is difficult due to an unstable aminal stereo-center that is sensitive to racemization, some asymmetric strategies have been developed to obtain pure (*S*)-enantiomers of DHQs (Table 10). Mainly, they consist of the cyclocondensation of anthranilamide and aldehydes in the presence of chiral catalysts that promote the formation of pure enantiomers, or the intramolecular amidation of *N*-Boc imines and anthranilamide (Scheme 11). The use of chiral phosphoric acidic catalysts is common in the enantioselective synthesis of DHQs, although they need a longer time (15–48 h) to complete the reaction. Various chiral phosphoric acids were employed to catalyze the cyclocondensation of anthranilamide and aldehydes,^240–243^ affording 67–99% yield and 26–99 ee%. An amidation of *N*-Boc imines and anthranilamide,^244,245^ gave pure (*S*)-enantiomer in 10–96 ee%. Scandium(iii)-catalytic systems were also effectively employed for the enantioselective cyclocondensation of anthranilamide and an aldehyde (Table 10).^246,247^

**Table 10:** Catalysts for the enantioselective synthesis of DHQs

Catalyst	Structure	Conditions	Time	% Yield	ee%	Ref.
TFA	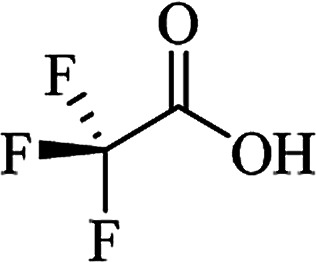	CH_3_CN, 45 °C	1.5 h	34–85	79–91	3
C_8_-TRIP	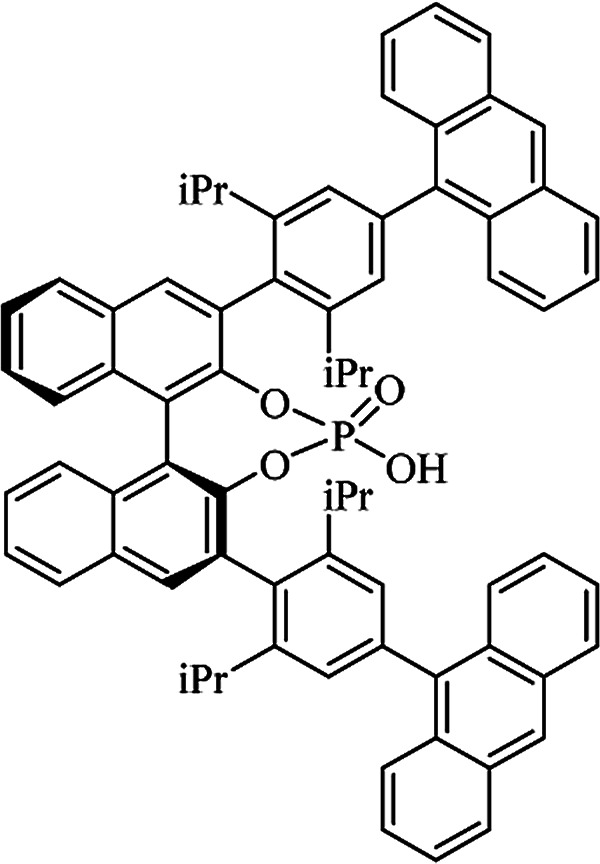	Toluene, 5 Å MS, −45 °C	24 h	67–94	26–98	240
9-Anthracenyl-TRIP	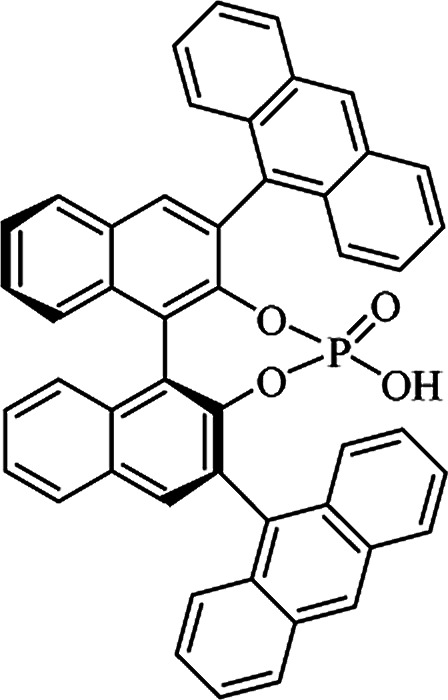	CHCl_3_, rt	24–48 h	73–99	90–99	241
		CHCl_3_, rt	24 h	30–94	10–96	244
(R,R)-PhDAP	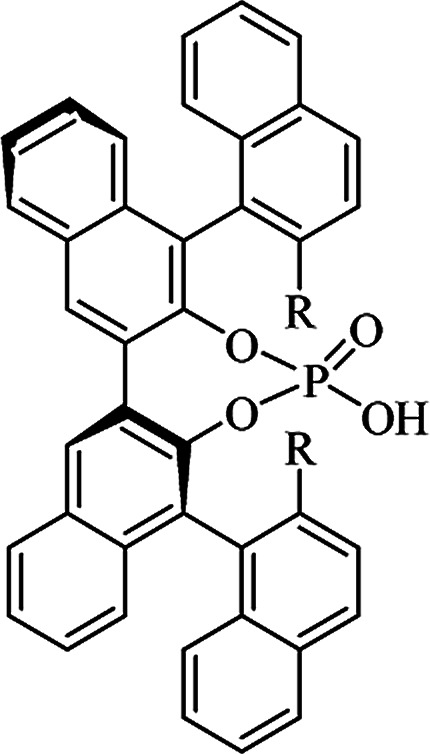	1,2-DFB, rt	15 h	99–100	80–86	242
9-Anthracenyl-SPINOL	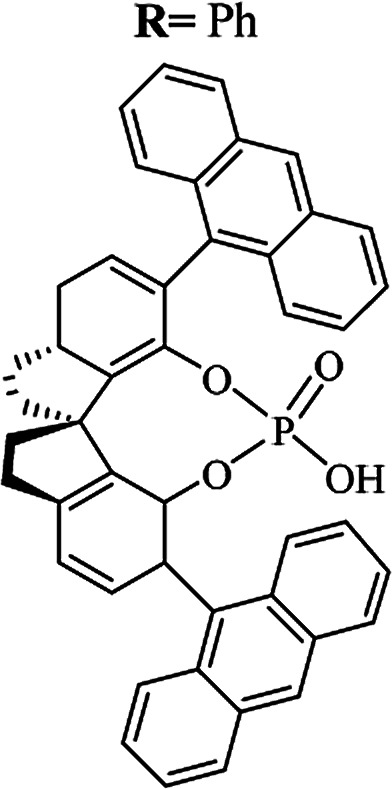	CHCl_3_, 3 Å MS, rt	24 h	88–99	59–98	243
BINOL-derived phosphoric acid	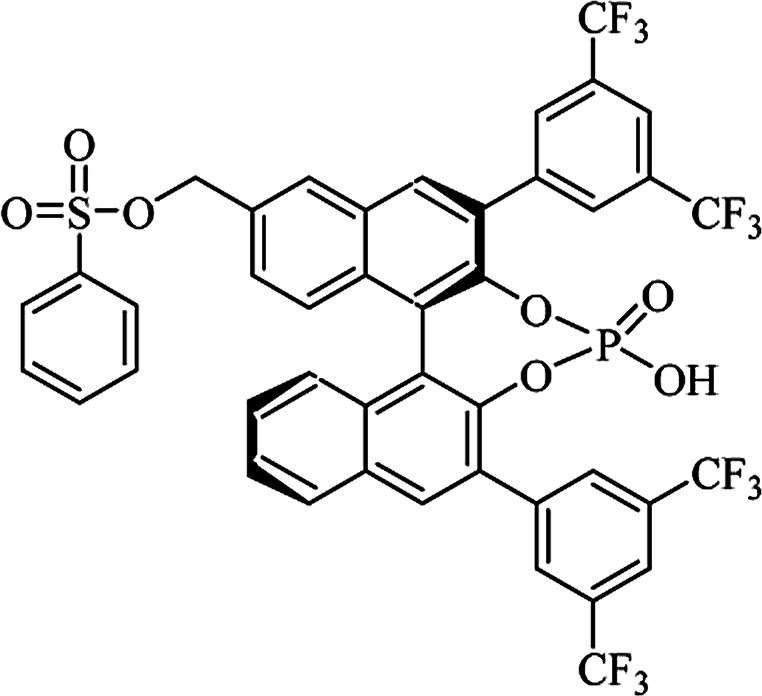	CHCl_3_, −15 °C	24–36 h	60–85	80–96	245
Sc(OTf)_3_/Pybox	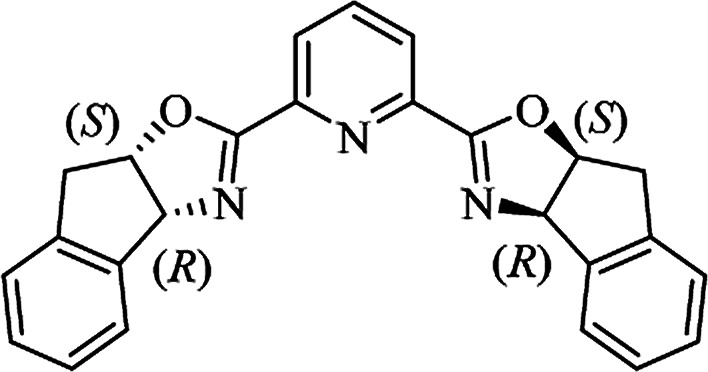	DCM, 4 Å MS, rt	6–48 h	80–94	86–98	246
Sc(OTf)_3_/L6	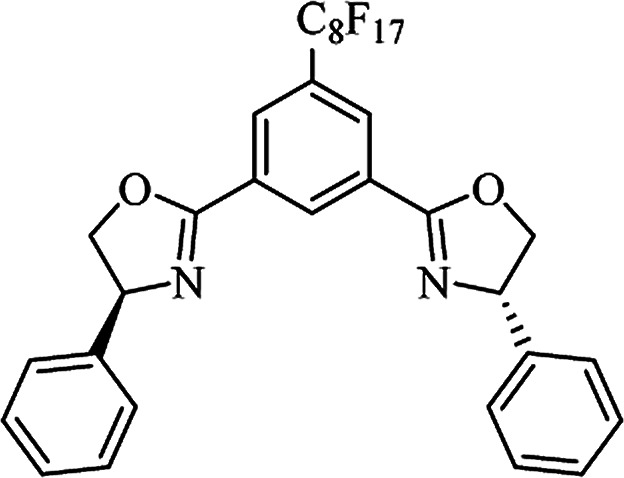	DCM, 4 Å MS, rt	48 h	81–94	87–98	247

### DHQ as an intermediate in organic chemistry

**Fig. 4:**
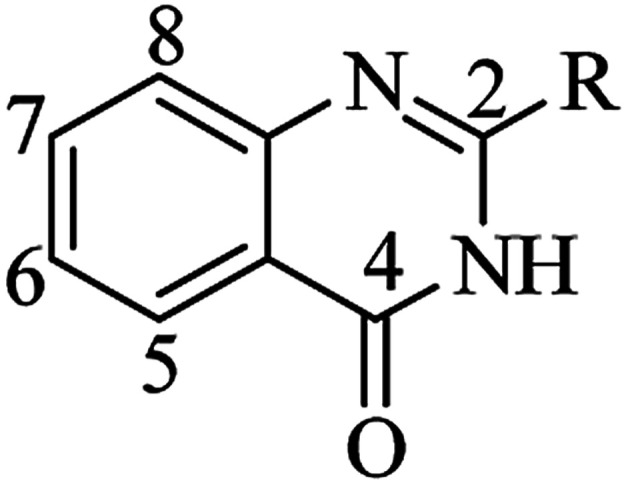
Quinazolin-4(3*H*)-one framework.

**Scheme 12:**
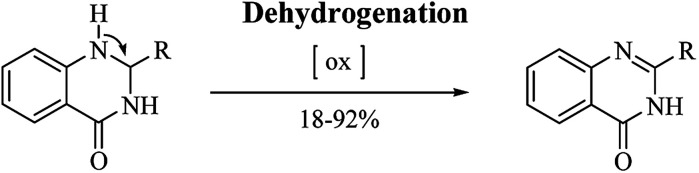
Dehydrogenation of DHQs as intermediate in organic synthesis.

In addition to their many significant pharmacological activities, DHQs also play a central role as intermediates in organic synthesis. In particular, they can be easily oxidized to the biologically active QZs. The quinazolinone ring (Fig. 4) is frequently encountered in organic chemistry as well as in medicinal chemistry.^248–254^ The QZ core is present in the structure of numerous natural products, especially alkaloids,^255,256^ and in some drugs,^257^ exhibiting various pharmacological properties. QZs also represent a privileged scaffold and many protocols are reported in literature for the synthesis of this important synthon. Among them, the dehydrogenation of DHQ derivatives (Scheme 12) has emerged as an easy and fast strategy to prepare QZs under different oxidant conditions (Table 11). Initially, QZs were obtained by dehydrogenation of DHQs by means of ZnCl_2_ in the presence of air, with 42% yield and in 10 h.^202^ A catalyst-free reaction in an open flask in refluxing EtOH was completed in few hours.^258^ The oxidation of DHQs by the addition of 2,3-dichloro-5,6-dicyano-1,4-benzoquinone also gave the corresponding oxidized derivatives with 83% yield.^231^ Various oxidizing agents were used for the dehydrogenation of DHQs to efficiently generate QZs, with 26–92% yield.^259–262^ Metal-catalyzed dehydrogenation was also attempted, affording the oxidized derivatives in moderate to good yields (18–85%) but in 16–24 h.^263–265^ Biocatalysis using laccase/*N*-hydroxybenzotriazole gave QZs in 62–87% yield.^266^

**Table 11:** Reaction conditions for the dehydrogenation of DHQs

Catalyst	Conditions	Time	% Yield	Ref.
ZnCl_2_	AcOH, air, reflux	10 h	42	202
Catalyst-free	EtOH, air, reflux	3 h	65	258
DDQ	MeOH, reflux	2 h	83.5	231
MnO_2_	DCM, rt	2 h	39–79	221
MnO_2_	CHCl_3_, rt	5–20 h	26–75	259
KMnO_4_	DMF, reflux	2–3 h	85–90	191
KMnO_4_	Acetone, rt	1 h		202
KMnO_4_	DMAC, MWI, 210 W	3–5 min	60–92	260
O_2_	AcOH, 150 °C	24 h	46–72	239
SO_2_	DMF/H_2_O, air or N_2_, 90 °C	5 h	65–92	261
K_2_S_2_O_8_	CH_3_CN, 90 °C	3–16 h	55–90	262
CuBr	K_2_CO_3_, DMSO, air, 130 °C	24 h	18–85	263
FeCl_3_	K_2_CO_3_, toluene, 120 °C	16 h	45–85	264
Ph_3_PAuNTf_2_	Toluene, 100 °C	24 h		265
Laccase/HBT	O_2_, citrate buffer (pH 4.5), 45 °C	20–30 h	62–87	266

### DHQ as a versatile fragment in drug design

**Fig. 5:**
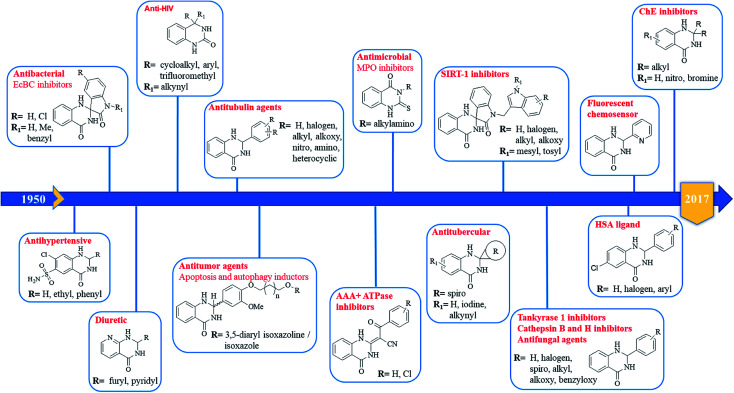
Pharmacological activities of DHQ derivatives.

The purpose of drug discovery has always been the design and development of “magic bullets” targeting a single key biomolecule in a central pathway of a specific disease. This led to the dominant paradigm “one target, one drug”, which might be inadequate to achieve a therapeutic effect for complex diseases.^267,268^ For this reason, the polypharmacology research and the design of multitarget compounds is considerably emerging,^269,270^ contributing to overcome some of the limitations of classical approach, in term of risks and costs.^271^ DHQ is a versatile fragment that can be easily functionalized at different positions. The introduction of specific moieties in the DHQ nucleus leads to the ability to interact with multiple targets, ensuring diverse pharmacological properties that make it a privileged scaffold (Fig. 5).

### 2-Aryl DHQ derivatives

The anticancer activity of DHQ derivatives was one of the first to be discovered. In 1967, Yale *et al.* identified DHQs as a new class of inhibitors of cell proliferation of the Earle's L cells. In particular, 2-aryl derivatives showed significant *in vitro* activity with low median effective dose (ED_50_) values of 0.1–6 μg ml^−1^.^13^ The antitumor activity of various 2-aryl DHQs against different cancer cell lines was then confirmed by several laboratories.^26,36,38,109,272^ Although many efforts to explain their cytotoxicity have been made, the target of the DHQ framework remains unknown. Almost three decades later, Hamel *et al.* used COMPARE algorithm to suggest that the antitumor effect of 2-aryl DHQs resulted from interactions with tubulin. Some derivatives inhibited the polymerization of tubulin at low micromolar concentrations and the binding of radiolabeled colchicine to tubulin at higher doses.^273^ Furthermore, based on the crystal structure of α,β-tubulin in complex with colchicine, through computational docking experiments and molecular dynamics, it was rationalized that (*S*)-DHQs may bind to tubulin better than the (*R*)-enantiomers, showing a better antitumor activity.^3^ In the same study, the accumulation of DHQs in the cytoplasm of MDA-MB-435 cells was observed through the inherent fluorescent properties of DHQs.^3^ In order to improve the antitumor effect of DHQs with antimitotic properties, a tumor-targeting liposomal delivery system that incorporates an anti-transferrin receptor single-chain antibody fragment was used, showing preferential targeting of tumor cells.^274^ 2-Quinolin-5-yl DHQ induced cytochrome *c* mediated apoptosis and autophagy in human leukemia MOLT-4 cells as demonstrated by flow cytometry, microscopy, LC3 immunofluorescence, and western blot analysis.^275^ Other pathways have been suggested to clarify the antitumor activity of DHQ derivatives. DHQs bearing a phenyl substituent and piperidine/piperazine moiety on C5 and C7 respectively were identified as selective inhibitors of p38 MAP kinase. They efficiently repressed the production of TNF-α in monocyte, THP-1 cells and LPS-stimulated whole blood (IC_50_ values in the nanomolar range). These analogs had good clearance but low oral bioavailability in rats. However, the introduction of a bulky *t*-butyl substituent on the piperidine nitrogen significantly increased the oral exposure in rats.^276^ Other DHQ derivatives were discovered by a high-throughput screening as inhibitors of the Hedgehog pathway, involved in embryonic development and oncogenesis. The biochemical mechanism of action of these DHQs was the inhibition of the AAA+ ATPase motor cytoplasmic dynein that converts chemical energy into mechanical force and regulates many cellular processes, including ciliary trafficking, formation of mitotic spindle and organelle transport. These AAA+ ATPase inhibitors could be useful to study cellular processes that require microtubule motor.^277^ Different targets were proposed to cause the antitumor activity of 2-aryl DHQs. Among them are cathepsin B and H, known to facilitate invasion, angiogenesis and metastasis through degradation of extracellular matrices and are associated with cancer progression.

Some 2-aryl DHQ derivatives were identified, through molecular docking studies, as reversible inhibitors of cathepsin B and H with effective inhibitory constant (*K*_*i*_) values in the nanomolar range.^278^ Through virtual and *in vitro* screening, 2-aryl DHQs were found to be potent tankyrase 2 inhibitors (IC_50_ ranging from low micromolar to nanomolar values). Crystal structures of tankyrase 2 with inhibitors showed that they interact with the nicotinamide-binding site of the catalytic domain of the enzyme. These compounds also inhibited Wnt/β-catenin pathway in a cell-based assay.^279^ Development of hybrid compounds that incorporate two pharmacophores in a single molecule with improved potency is gaining momentum. A new class of hybrids was designed and synthesized from DHQ and 3,5-diaryl isoxazoline/isoxazoles, linked through alkane spacers, and their antitumor activity was evaluated. Derivatives with 3C chain spacers showed excellent potency against lung cancer, exhibiting nanomolar IC_50_ values. Flow cytometric analysis revealed G2/M cell cycle arrest, usually associated with the inhibition of tubulin polymerization. Furthermore, these compounds disrupt microtubules, inhibit cyclin B1, CDK1, and induce apoptosis in cancer cells.^280^*N*-indolylmethyl substituted spiroindoline-3,2′-DHQ were hypothesized as potential sirtuin inhibitors. Sirtuins, whose family consists of seven members (SIRT1-7), are important targets for cancer therapy, being up-regulated in several types of cancer. In particular, SIRT1 has several substrates, including p53 and NF-kB, and its inhibition leads to the re-expression of silenced tumor suppressor genes and the subsequent decrease of cancer cell growth. These new DHQ derivatives were obtained through Pd/C–Cu-mediated coupling cyclization and tested *in vitro* using a yeast homologue of mammalian SIRT1, Sir 2 protein, showing dose-dependent inhibition. Molecular docking analysis showed that the benzene ring of the DHQ occupied the deep hydrophobic pocket of the protein, while the NH and the sulfonyl groups form H-bonding interaction with select amino acid residues (Val412 and Gly415).^281^ Over half a century ago, DHQ derivatives bearing a sulfonamide moiety on C7 showed diuretic activity, causing natriuresis and chloruresis and a slight increase of potassium excretion after oral administration.^282^ Among them, fenquizone is a FDA-approved drug for the treatment of edema and hypertension.^283–286^ The substitution of C8 with N in the benzene ring of DHQ and the introduction of a pyridyl moiety on C2 led to 1,2-dihydro-2-(3-pyridyl)-3*H*-pyrido[2,3-*d*]pyrimidin-4-one derivatives. They also showed diuretic activity.^287^ Some DHQ derivatives also show broad-spectrum antimicrobial properties. 2-(5-Nitro-2-thienyl) DHQs showed antibacterial activity against *Hemophilus vaginalis* and *Escherichia coli* strains, responsible for bacterial vaginitis, exhibiting low minimal inhibitory concentration (MIC, ranging from 0.4 to 12.5 μg ml^−1^).^288^ More recently, some spiro-oxindole DHQs have shown significant antibacterial activity against both Gram-positive and Gram-negative bacterial strains, in particular *E. coli*. In order to explain the mechanism of this antibacterial activity, DHQs were docked on *E. coli* biotin carboxylase (EcBC) enzyme. EcBC is a known target of the fatty acid biosynthetic pathway since it catalyzes the ATP-dependent carboxylation of the vitamin biotin. Molecular docking results showed that the DHQ core fit in the hydrophobic enclosure formed by Ile437 and His236, and the different substituents form H-bonds as well as hydrophobic interactions with specific amino acid residues.^289^

Tuberculosis, an infectious disease caused by *Mycobacterium tuberculosis*, is a worldwide leading cause of death. Multiple antibiotics are needed to treat tuberculosis over a long period of time, but the development of multiple drug-resistant tuberculosis limits complete recovery. Some spiro-DHQ derivatives were tested for their *in vitro* inhibitory activity against *Mycobacterium tuberculosis* H37Rv chorismite mutase, a promising target for the identification of new antitubercular drugs. These derivatives showed a moderate inhibition at relatively high doses (30 μM).^290^ 2-Aryl DHQs also show moderate to good antifungal activities, in particular against *Candida albicans* and *Aspergillus niger*.^43^ A class of 2-thioxo DHQ derivatives showed an excellent antimicrobial activity by inhibiting myeloperoxidase. This enzyme plays an important role in host defense and contributes to inflammation. They reversibly inhibited myeloperoxidase, exhibiting IC_50_ values in the nanomolar range.^291^ 4-Alkynyl-3,4-dihydro-2(1*H*)-quinazolinones, bearing a second substituent at position 4, such as cycloalkyl, aryl and trifluoromethyl were identified as potent HIV-1 non-nucleoside reverse transcriptase inhibitors, inhibiting wild-type and various mutant forms of HIV-1. These compounds also showed a good oral bioavailability.^292–294^ 2-Aryl DHQ derivatives, with or without substituents on N1 and N3, were found to interact with human serum albumin (HSA), using fluorescence spectroscopy. The therapeutic effects of drugs depend on their absorption, distribution, metabolism and excretion, and can be influenced by the binding affinities of drugs with HSA. In particular, strong binding can reduce free drug concentrations in plasma whereas weak binding can decrease lifetime and/or distribution of drugs. Results of site marker competitive experiments revealed that DHQs spontaneously bind to HSA on site II, subdomain IIIA, though hydrophobic forces. Various substituents in the benzene ring of DHQ could increase the interactions with HSA, forming additional van der Waals forces and H-bonds.^295–297^ Transition metals, such as iron, zinc and copper, play important roles in the human body. Specifically, copper is a catalytic cofactor for a variety of metalloenzymes and physiological processes. Increased levels of copper in the body could be involved in the production of reactive oxygen species (ROS), causing imbalance in cellular functions and several diseases. For this reason, the development of fluorescent chemosensors for biologically active transition metal ions are becoming attractive. In this regard, 2-aryl DHQs were efficiently used as fluorescent probes to selectively detect Cu^2+^ ions.^298^

A series of 2-disubstituted DHQ derivatives showed inhibitory activity against cholinesterases (ChE), involved in the lysis of choline-based esters that act as neurotransmitters. Acetylcholinesterase is present in chemical synapses and in red blood cell membranes, and butyrylcholinesterase is found in blood plasma. ChE inhibitors are the only effective therapeutic approach for the symptomatic treatment of Alzheimer's disease. These derivatives inhibited both enzymes (IC_50_ values in micromolar range), better than or comparable to the standard drug galantamine. Molecular docking studies revealed that the benzene ring of DHQs can fits into the choline-binding site while the phenyl ring at position 2 is oriented towards the peripheral anionic site. The NH group of DHQs and Asp72 form an ion-dipole interaction, while substituents in the benzene ring are involved in H-bonding interactions.^16^

### Mono- and di-substituted DHQ derivatives

As a privileged scaffold, the DHQ nucleus is amenable to modification. The most common functionalization was the single introduction of different chemical groups on heterocyclic N1 and mainly on N3, and substitutions on both positions. The resulting mono- and di-substituted derivatives showed specific activities depending on the substituents (Table 12).

**Table 12:** Mono- and di-substituted DHQs

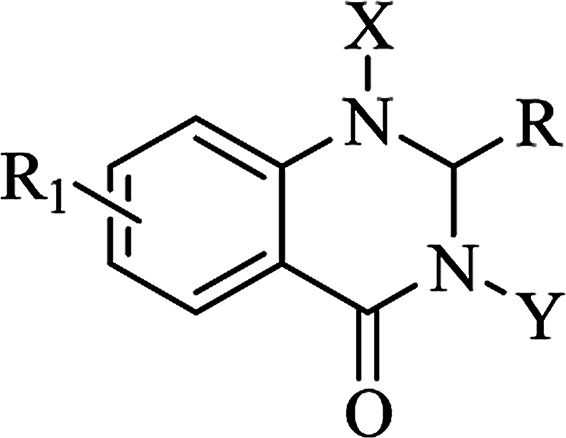					
R	X	Y	R_1_	Activity/mechanism	Ref.
Alkyl, cycloalkyl	Phenyl, benzyl	H	H, alkyl, alkoxy, amino, nitro	Anti-inflammatory	298 and 299
Dimethyl	Alkyl, cycloalkyl, aryl	H	Heterocycles	PKCθ inhibitors	301
H	Phenyl	H	Amide, alkylamino, sulfonamine	p38 MAPK inhibitors	302
H, phenyl, furyl, phenylamino	H	Alkylamino, cycloalkylamino, hydroxy, alkoxy	H	Antibacterial	311 and 312
Phenyl	H	Alkylamino	H	Antimalarial	313
Thienyl, furyl, pyrrolidinyl	H	Phenyl	H	Antiviral	314 and 315
Thienyl, pyridinyl, indolyl	H	Phenyl	H	Antiprotozoal/shiga toxin	316
Phenyl	H	Furan-2-ylmethyl, benzyl	H, hydroxy, methoxy	TSHR inhibitors	45 and 305
Alkyl	H	Biphenyl with *o*-tetrazole	H, alkoxy	Angiotensin II receptor antagonists	234
Alky, spiro	H	Alkyl	Phenyl, benzyloxy	Antifungal/lysozyme	317
Pyridinyl-1*H*-pyrazolyl	H	Alkyl, cycloalkyl	H, alkyl, halo	Insecticidal/calcium channels	318
Alkyl, phenyl	H	Alkyl	H, alkyl	Anticonvulsant/Na^+^/Ca^2+^ exchanger inhibitors	230 and 319
H	H	Heterocycles	H, heterocycles	CDK5 inhibitors	320
H	H	Heterocycles	H	M_1_ and M_4_ receptors agonists	321
Keto	H	2-Oxoindolinyl	H	Antitumor	322
H	H	Phenyl	Amino, phenyl	Antitumor/p38 MAPK inhibitors	323
Keto, spiro	H	Alkyl, aryl	Methoxy, oxazolyl	IMPDH II inhibitors	324
H, methyl	Acyl, alkyl, phenyl	Aryl	H	Analgesic and anti-inflammatory	300 and 326
H	Acyl	Alkyl, phenyl	H	Choleretic and antifibrillatory	327

**Scheme 13:**
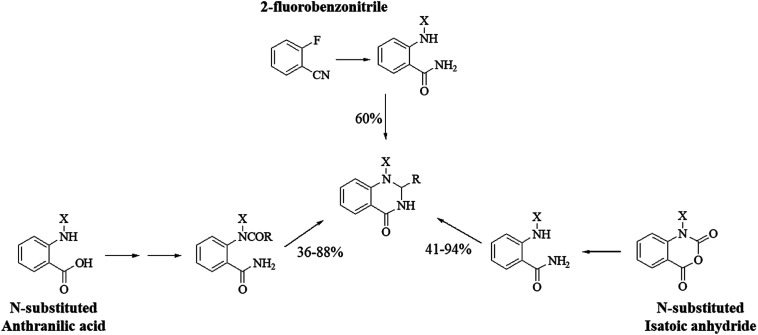
Synthetic strategies for 1-substituted DHQ derivatives.

1-Substituted DHQs can be synthesized similarly to the unsubstituted derivatives, starting from anthranilic acid^284^ and isatoic anhydride,^299,300^ bearing substituents on the amino group and the heterocyclic N respectively, or 2-fluorobenzonitrile (Scheme 13).^301^ Most DHQ derivatives bearing aryl substituents on N1 showed good anti-inflammatory properties.^298,299^ Some 1-(2-phenylethyl) DHQs were found to inhibit IL-2 production through inhibition of the protein kinase C-θ (PKCθ),^301^ while 1-aryl-6-substituted DHQs inhibited p38 *in vitro* at nanomolar concentrations.^302^

**Scheme 14:**
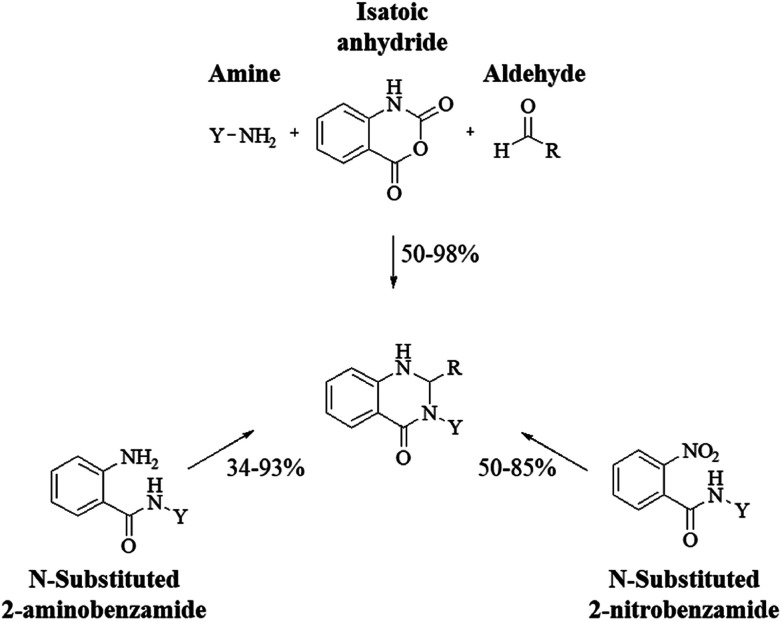
Synthetic strategies for 3-substituted DHQ derivatives.

More commonly, 3-substituted DHQs were synthesized by cyclocondensation of 2-aminobenzamides,^303–305^ or 2-nitrobenzamides,^306^ substituted on the amide groups, with different aldehydes. 3-substituted DHQs could be also obtained through the one-pot three-component reaction of isatoic anhydride with amines and aldehydes (Scheme 14).^307–310^ DHQ derivatives bearing cyclic amino substituents on N3 showed antibacterial activity,^311,312^ while 3-alkylamino DHQs exhibited antimalarial effects.^313^ Different heterocycles like thienyl, furyl and pyrrolidinyl in position 2 or 3-phenyl DHQs were responsible for antiviral activity,^314,315^ and for the protective effect against Shiga toxin.^316^ 3-(Furan-2-ylmethyl) derivatives were reported as thyroid-stimulating hormone receptor agonists.^45,305^ 3-Biphenyl substituted DHQs with a tetrazole ring in the *ortho* position acted as angiotensin II receptor antagonists.^234^ 3-Alkyl substituted DHQs bind to lysozyme showing antifungal activity.^317^ Certain 3-alkyl and 3-cycloalkyl DHQ derivatives exhibit insecticidal properties by targeting calcium channels.^318^ On the other hand, 3-alkyl and 3-alkylamino substituted 3,4-dihydro-2(1*H*)quinazolinones show anticonvulsant activity^230^ and inhibited the Na^+^/Ca^2+^ exchanger.^319^ Other 3,4-dihydro-2(1*H*)quinazolinones bearing heterocyclic substituents at position 3 showed neuroprotective and antipsychotic properties, inhibiting cyclin-dependent kinase 5 (CDK5),^320^ and targeting M_1_ and M_4_ muscarinic acetylcholine receptors,^321^ respectively. 3-(2-Oxoindolin-3-yl) DHQ derivatives showed antiproliferative activity against human tumor cell lines (IC_50_ in the micromolar range).^322^ Other 3-aryl and 3-alkyl DHQs also showed antitumor properties, inhibiting p38 MAP kinase,^323^ and inosine 5′-monophospate dehydrogenase type II (IMPDH II).^324^

**Scheme 15:**
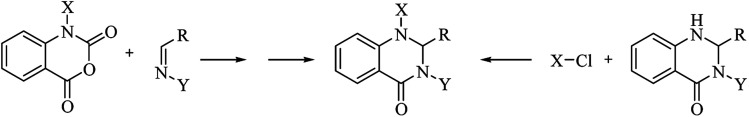
Synthetic strategies for 1,3-disubstituted DHQ derivatives.

The less studied 1,3-disubstituted DHQs could be obtained in two different ways: through the cyclization of *N*-substituted isatoic anhydride and substituted arylidene anilines,^299^ or the reaction of 3-substituted DHQ with chloride (Scheme 15).^325^ 1,3-Disubstituted DHQ derivatives mostly showed anti-analgesic and inflammatory activities.^300,326^ 1-Acyl DHQs bearing alkyl and phenyl substituents on N showed choleretic and antifibrillatory activities.^327^

## Conclusions

The importance of the DHQ nucleus has emerged due to its versatility as suitable substrate for functionalization and its remarkable bioactivities. Many procedures have been reported for the synthesis of DHQ derivatives as racemic mixture. Although the cyclocondensation of anthranilamide and an aldehyde and the one-pot three-component reaction of isatoic anhydride, ammonium acetate and an aldehyde represent the most common ways to obtain DHQ derivatives, several other methods have been suggested. Many approaches have been investigated, from classical to greener and more sustainable reaction conditions. Recently, enantioselective strategies have been attempted in order to obtain pure (*S*)-enantiomers. Furthermore, the DHQ scaffold is an important intermediate in organic chemistry and can easily be oxidized into QZ scaffold. On the other hand, various important bioactivities have been associated to the DHQ scaffold and reported in the literature. On this basis, the DHQ nucleus imposes itself as a privileged scaffold in drug design and an interesting fragment for drug discovery.

## Conflicts of interest

There are no conflicts to declare.

## Supplementary Material
